# On the Potential Role of Phytate Against Neurodegeneration: It Protects Against Fe^3+^-Catalyzed Degradation of Dopamine and Ascorbate and Against Fe^3+^-Induced Protein Aggregation

**DOI:** 10.3390/ijms26104799

**Published:** 2025-05-16

**Authors:** Samantha Rebeca Godoy, Pilar Sanchis, Juan Frau, Bartolomé Vilanova, Miquel Adrover

**Affiliations:** 1 Interdisciplinary Group on Neurodegeneration, Vascular and Metabolic Diseases (INNoVAM), Departament de Química, Universitat de les Illes Balears, Ctra. Valldemossa km 7.5, E-07122 Palma de Mallorca, Spain; s.godoy@uib.cat (S.R.G.); pilar.sanchis@uib.es (P.S.); juan.frau@uib.es (J.F.); bartomeu.vilanova@uib.es (B.V.); 2 Health Research Institute of the Balearic Islands (IdISBa), Ctra. Valldemossa 79, E-07010 Palma de Mallorca, Spain; 3 Institut Universitari d’Investigació en Ciències de la Salut (IUNICS), Universitat de les Illes Balears, Ctra. Valldemossa km 7.5, E-07122 Palma de Mallorca, Spain; 4 CIBER Fisiopatología de la Obesidad y Nutrición (CIBERObn), Instituto de Salud Carlos III, 28029 Madrid, Spain

**Keywords:** dopamine, ascorbic acid, phytic acid, α-synuclein

## Abstract

Myo-inositol-1,2,3,4,5,6-hexakisphosphate (IP6) is commonly found in plant-derived foods and has important pharmacological properties against many pathologies. One of them appears to be neurodegeneration, which is notably stimulated by dysregulated metal metabolism. Consequently, we explore the role of IP6 in mitigating neurodegenerative events catalyzed by dysregulated free iron. More precisely, we performed spectrophotometric measurements in aqueous solutions to investigate the ability of IP6 to chelate Fe^3+^ and inhibit its role in catalyzing the oxidative degradation of dopamine and ascorbic acid, two key molecules in neuronal redox systems. Our results demonstrate that IP6 effectively prevents the formation of harmful intermediates, such as neuromelanin and reactive oxygen species, which are linked to neuronal damage. Additionally, we assessed the effect of IP6 on Fe^3+^-induced protein aggregation, focusing on α-synuclein, which is closely associated with Parkinson’s disease. Our data reveal that IP6 accelerates the conversion of toxic α-synuclein oligomers into less harmful amyloid fibrils, thereby reducing their neurotoxic potential. Our findings highlight the dual function of IP6 as a potent Fe^3+^ chelator and modulator of protein aggregation pathways, reinforcing its potential as a neuroprotective agent. Consequently, IP6 offers promising therapeutic potential for mitigating the progression of neurodegenerative disorders such as Parkinson’s and Alzheimer’s diseases.

## 1. Introduction

Myo-inositol-1,2,3,4,5,6-hexakisphosphate, also known as phytic acid or phytate (IP6), is the main phosphorus reservoir in most legumes, oilseeds, and whole grains [1]. During germination, phytases hydrolyze IP6 to release phosphate, which is essential for plant growth and development. Consequently, the IP6 content in these seeds ranges from approximately 0.05% to 6.5% of their overall weight [2]. Therefore, the consumption of legumes and cereals accounts for 13% and 77%, respectively, of the total human intake of IP6 (~0.2–1 g/day) [3,4]. Once ingested, IP6 is primarily absorbed in the stomach, upper intestine, and colon [4]. However, its bioavailability is relatively low due to the limited presence of phytases in the digestive tract and its highly negative charge, which makes crossing the cellular lipid bilayer difficult [5]. Consequently, most of the ingested IP6 is excreted [4]. Nonetheless, a portion of the absorbed fraction may enter cells via pinocytosis [6].

Absorbed IP6 exhibits numerous physiological roles. IP6 and its lower inositol phosphates appear to play distinct roles in signal transduction processes by modulating enzymatic activity and interacting with specific proteins, particularly those involved in regulating intracellular calcium levels [7] and those associated with membrane trafficking events, such as synaptotagmin [8] and arrestins [9]. Additionally, IP6 and other inositol phosphates are implicated in DNA repair mechanisms through the activation of DNA-dependent protein kinases [10], as well as in the regulation of nuclear mRNA transport [11]. Moreover, it is suggested that IP6 may also influence digestion and nutrient absorption. Evidence indicates that IP6 inhibits enzymes such as amylase, pepsin, and trypsin, potentially reducing the digestibility of carbohydrates and proteins [12,13]. Furthermore, due to its strong negative charge, IP6 can chelate cations such as Zn^2+^, Ca^2+^, Mg^2+^, Mn^2+^, Cu^2+^, and Fe^3+^, thereby reducing the absorption of minerals and trace elements [14]. However, this anti-nutritional effect has only been observed in vitro; data from in vivo experiments do not support mineral deficiencies associated with IP6 consumption [15,16].

Beyond its physiological role, IP6 is also regarded as a powerful nutraceutical, with significant potential to treat or prevent a wide range of disorders through various mechanisms of action. Its high affinity for the Ca^2+^ cation integrated into hydroxyapatite (HAP) seeds makes it a potent inhibitor of HAP growth and, consequently, in vivo calcifications. IP6 not only blocks the propagation of growth steps on the surface of HAP crystals but also prevents the agglomeration of HAP clusters into large entities that eventually form HAP concretions [17]. As a result, IP6 can prevent vascular calcification in patients undergoing hemodialysis [18], and its consumption is inversely associated with aortic calcification [19]. Since most kidney stones are composed of calcium oxalate, calcium phosphate, or a mixture of both, IP6 can also inhibit calcification in the kidneys and papillae [20]. In fact, a diet rich in IP6 prevents renal calculi [21], while an IP6-free diet leads to nephrocalcinosis [22]. Furthermore, IP6 can reduce calcified dental plaque [23]. The adsorption of IP6 to preformed crystal faces can also inhibit their dissolution. This explains why IP6 can prevent bone decalcification [24,25] and, consequently, osteoporosis [25,26].

The interaction of IP6 with Ca^2+^-based crystallizations is not the only mechanism through which IP6 exerts its beneficial effects. IP6 also appears to have positive effects on patients with type 2 diabetes. This may be related to the fact that alterations in inositol metabolism are associated with hyperglycemia and insulin resistance [27]. Furthermore, IP6 inhibits amylase, reducing the rate of carbohydrate hydrolysis and absorption [28,29]. It also decreases leptin levels, thereby promoting satiety and energy utilization, while increasing adiponectin levels, which stimulates the antioxidant response [28,30,31]. In addition, IP6 reduces lipase activity, total cholesterol, LDL cholesterol, and hepatic total lipid levels, while increasing HDL cholesterol levels [27,32]. Moreover, IP6 can inhibit the formation of Fe^3+^-catalyzed advanced glycation end products [33]. These products seem to play a critical role in the development of diabetes-related complications. This aligns with evidence showing that a diet rich in legumes or whole grains reduces glycated hemoglobin (HbA1c) levels [34,35]. It is also well-established that IP6 possesses significant anticancer properties, as it can inhibit molecular pathways involved in tumor progression, including metastasis, angiogenesis, apoptosis, and differentiation [36]. Its potential against cancer progression is demonstrated in cases of colon [37], lung [38], and breast [39] cancers. Additionally, IP6 exhibits antibacterial [40] and antiviral [41] properties.

Numerous experimental data indicate that IP6 plays a crucial role in the normal functioning of the central nervous system. Its concentration in the brain (~2 μg IP6/g) is approximately 10 times higher than that in the kidneys, liver, and bones [42,43], and it is evenly distributed across different brain regions. Within the brain, it is involved in vesicle trafficking [44] and neurotransmitter release [45]. Furthermore, it binds with high affinity to specific proteins, such as myelin proteolipid protein [46] and L-type Ca^2+^ channels [7]. Moreover, neuronal IP6-dependent protein kinases have been identified, including those that phosphorylate pacsin/syndapin I, which are involved in synaptic vesicle (SV) recycling [47].

Furthermore, IP6 may play a protective role against neurodegeneration. Diets rich in IP6 have been positively associated with better cognitive function in elderly individuals [48], a slower rate of cognitive decline [49], and improved academic performance in children [50]. Specifically, IP6 (30–100 μM) increases the viability of mesencephalic cells by 19–42% [51]; suppresses dopaminergic neuron loss in the substantia nigra by 55% [52]; protects rat dopaminergic cells (N27) from apoptosis [53]; and shields human dopaminergic cells (SH-SY5Y) against lipid peroxidation, reactive oxygen species (ROS) production, and the accumulation of α-synuclein (αS), a hallmark of Parkinson’s disease [54]. Moreover, IP6 demonstrates inhibitory activity against BACE1, potentially preventing the accumulation of amyloid-beta, a hallmark of Alzheimer’s disease [55].

Part of the protective effect of IP6 against neurodegeneration may be attributed to its ability to (i) reduce ROS in peripheral neuropathy [56]; (ii) attenuate inflammatory responses in Parkinson’s disease mouse models [52]; (iii) reduce apoptosis by stimulating autophagy [2,48] and decreasing caspase-3 activity [51]; and (iv) restore mitochondrial membrane potential, a critical factor for neuronal survival [56]. However, its main mechanism of action is attributed to its ability to bind iron [57,58]. Iron homeostasis plays a pivotal role in brain health, as iron serves as a cofactor for enzymes involved in neurotransmission. Consequently, its dysregulation is a hallmark of many neurodegenerative diseases. In Alzheimer’s disease, abnormal iron accumulation is detected in the hippocampus and cortex, regions critical for memory and cognition [59]. Iron promotes amyloid-beta peptide aggregation and enhances neurotoxicity by catalyzing ROS production through Fenton reactions, leading to pro-inflammatory responses. Similarly, in Parkinson’s disease [60], elevated iron levels in the substantia nigra contribute to oxidative damage and αS aggregation, two key pathological features. Additionally, in Huntington’s disease and amyotrophic lateral sclerosis, iron-induced oxidative stress is clearly implicated in neuronal loss [61].

Abnormal accumulation of free iron can also induce neurodegeneration through the degradation of key molecules essential for normal brain function, such as dopamine (DA) [62] or ascorbic acid (AA) [63]. DA is critical for neurotransmission [64], while AA serves as a key antioxidant in neurons [65,66]. Given that IP6 can chelate Fe^3+^ [57,58], we applied UV-Vis and fluorescence spectroscopies to investigate its potential to inhibit Fe^3+^-catalyzed degradation of DA and AA in aqueous solutions. Additionally, we examined whether IP6 could prevent Fe^3+^-catalyzed aggregation of proteins linked to neurodegenerative diseases, such as αS in Parkinson’s disease. Our results demonstrate that IP6 plays a protective role against neurodegeneration by inhibiting harmful molecular processes mediated by dysregulated free iron.

## 2. Results

### 2.1. Understanding the Environmental Factors Affecting the Degradation of DA

#### 2.1.1. The Conversion of AC into NM Is Dependent on the Initial Concentration of DA

**Figure 1:**
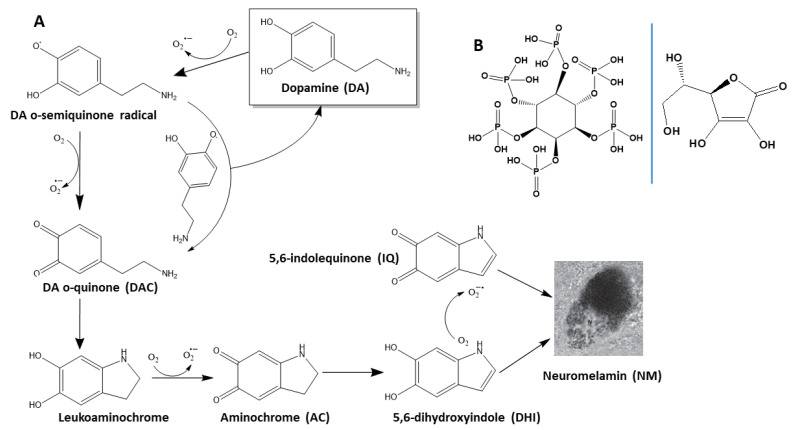
Oxidation pathway of DA to form NM and structural formula of IP6 and AA. (**A**) Chemical structures of the products generated during DA oxidation. The non-enzymatic degradation of DA produces superoxide radicals and quinones, which subsequently aggregate to form insoluble neuromelamin (NM). (**B**) Chemical structures of myo-inositol-1,2,3,4,5,6-hexakisphosphate (phytate; IP6; **left**) and ascorbic acid (AA; **right**).

DA that is not entrapped in dopaminergic vesicles undergoes spontaneous oxidation. This process begins with the loss of one electron, forming a DA o-semiquinone radical, accompanied by the reduction of one O_2_ molecule to a superoxide radical (O_2_·^−^). The reaction between two DA o-semiquinone radicals produces one molecule of DA and one molecule of DA o-quinone (DAC). Alternatively, the DA o-semiquinone radical can transfer an electron to another O_2_ molecule, forming DAC, which is stable only at a pH < 2. Under physiological conditions, DAC rapidly cyclizes to leukoaminochrome, which subsequently oxidizes to aminochrome (AC). Finally, AC rearranges into 5,6-dihydroxyindole (DHI), which later oxidizes to form 5,6-indolequinone (IQ). Both IQ and DHI co-polymerize through π-stacking interactions to generate neuromelanin (NM) (Figure 1A). The conversion of DA to NM can be monitored by measuring the absorbance increase at 475 nm (attributed to AC formation; ε_475nm_ = 3245 M^−1^ cm^−1^ [67]) or at 650 nm, which reflects NM polymerization without interference from other intermediates [68]. DA, DAC, and DHI do not significantly affect AC measurements, as most phenolic compounds primarily absorb at ~290 nm [67,69].

To investigate how different environmental conditions influence DA degradation, we used UV-Vis spectroscopy. The UV-Vis spectrum of DA exhibited temporal changes upon incubation, following a concentration-dependent mechanism. At DA concentrations ranging from 30 to 100 μM, a gradual appearance of the characteristic band for AC at 475 nm was observed. Interestingly, once formed, AC remained relatively stable in solution, as its spectral band did not diminish over time. This stability suggests that DA does not rapidly progress to DHI or IQ. However, the formation of small amounts of NM was evident, as indicated by increased absorbance at 650 nm (Figure S1A–C) and visual detection of insoluble black particles (Figure S2). Notably, at DA concentrations >500 μM, NM formation occurred at a significantly faster rate and in larger quantities. Under these conditions, AC rapidly progressed toward NM polymerization, evidenced by the disappearance of the 475 nm band within 20 min of incubation (Figure S1D,E).

These results demonstrate that under aerobic conditions and physiological pH, DA degrades to form AC, which subsequently initiates NM polymerization. However, the rate and yield of NM formation are directly dependent on DA concentration.

#### 2.1.2. Phosphate Catalyzes the Formation of NM from of AC

Next, we investigated the effect of phosphate—a component present under physiological conditions—on DA degradation. For this study, a 100 μM DA solution was used, enabling independent observation of AC and NM formation (Figure S1C). In the absence of phosphate, AC was formed alongside other phenolic compounds, as indicated by a spectral band at ~300 nm. However, none of these compounds progressed toward NM formation, evidenced by the lack of absorbance changes at 650 nm over time (Figure S3A). When 1 mM phosphate was present, NM formation remained undetectable, but a slight increase in AC and phenol compounds was observed after 120 min of incubation (Figure S3B). At 10 mM of phosphate, AC formation was stimulated, inducing tiny amounts of NM (Figure S3C). At concentrations > 100 mM of phosphate, DA rapidly polymerized into NM, with the characteristic spectroscopic bands of AC and phenolic compounds becoming undetectable (Figure S3D,E).

These findings demonstrate that phosphate accelerates DA degradation into AC and other polyphenols, further confirming its essential role in NM polymerization.

#### 2.1.3. NaCl Slightly Affects the Degradation Rate of DA

Given that NaCl is the most abundant salt in the intracellular space, we examined its effects on DA degradation. Temporal UV-Vis spectral changes were monitored for a 100 μM DA solution prepared in 10 mM phosphate buffer with 0, 20, 75, or 300 mM NaCl. Adding 20 mM NaCl slightly enhanced AC and NM formation. Increasing NaCl to 75 mM showed a minimal effect on AC/NM production. A pronounced catalytic effect emerged only at 300 mM NaCl (Figure S4), accelerating both AC formation and NM polymerization. These results demonstrate that physiologically relevant NaCl concentrations (≤75 mM) have negligible catalytic effects on DA degradation. Significant NM polymerization catalysis by NaCl requires concentrations that exceed typical intracellular levels.

#### 2.1.4. On the Effect of Oxidants and Reductants on the Degradation of DA

Under oxidizing conditions, DA rapidly degrades, forming the products shown in Figure 1A. While NM is formed through π-stacking interactions between IQ and DHI, most DA degradation products arise directly from its oxidation. To explore this further, we investigated whether the addition of strong oxidants (e.g., H_2_O_2_ and KIO_3_) accelerated the DA oxidation rate and/or increased the yield of degradation products.

The temporal variation in the UV-Vis spectra of DA showed no significant changes after adding H_2_O_2_ or KIO_3_. The only notable difference was a slight absorbance increase at ~650 nm, indicating modest NM production enhancement (Figure S5A–C). This suggests that the oxidizing capacities of H_2_O_2_ and KIO_3_ (at 50 μM concentrations) are negligible compared to that of O_2_. Likely explanations include their similar standard redox potentials (Er^0^: H_2_O_2_/H_2_O ~ 1.7 V; IO_3_^−^/I^−^ ~ 1.2 V; O_2_/H_2_O ~ 1.2 V) and the substantially higher dissolved O_2_ concentration in the phosphate buffer (~250 μM) [70].

Since DA degradation occurs via a redox process, the addition of reductants would be expected to inhibit DA oxidation. Indeed, 50 μM AA or TCEP suppressed all temporal changes in the UV-Vis spectrum of DA (Figure S5D,E), confirming their efficacy as DA oxidation inhibitors. This aligns with the high neuronal AA concentration (~10 mM) [65], suggesting its critical role in the antioxidant machinery to prevent cytoplasmic free DA oxidation.

Our findings demonstrate that under aerobic conditions, oxidants do not significantly enhance DA oxidation rates, whereas reducing agents such as AA completely inhibit DA degradation.

#### 2.1.5. Fe^3+^ Enhances the Degradation Rate of DA

**Figure 2:**
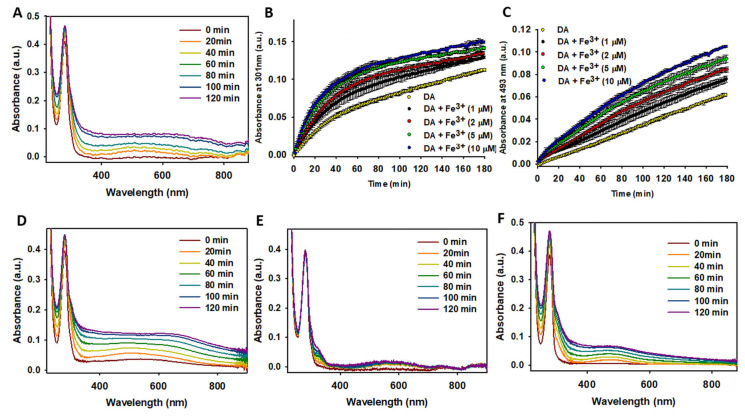
Study of the effect of Fe^3+^ on DA degradation. (**A**) Temporal variation of UV-Vis absorbance spectra for a solution containing DA (100 μM) in the presence of Fe^3+^ (5 μM). (**B**,**C**) Temporal absorbance variation at 301 nm (**B**) and at 493 nm (**C**) of a solution containing DA (100 μM) in the absence (yellow dots) and presence of Fe^3+^ at different concentrations: black dots (1 μM), red dots (2 μM), green dots (5 μM), and blue dots (10 μM). (**D**) Temporal variation of UV-Vis spectra of a solution containing DA (100 μM) in the presence of Fe^3+^ (5 μM) and H_2_O_2_ (50 μM). (**E**) Temporal variation of UV-Vis spectra of a solution containing DA (100 μM) in the presence of Fe^3+^ (5 μM) and tris(2-carboxyethyl)phosphine (TCEP; 50 μM). (**F**) Temporal variation of UV-Vis spectra of a solution containing DA (100 μM) in the presence of Fe^3+^ (5 μM) and EDTA (50 μM). All solutions were prepared in 10 mM phosphate (pH 7.4) containing 75 mM NaCl, and spectra were recorded at 37 °C. Statistics: Data in panels (**B**,**C**) (*n* = 3) were analyzed using two-way repeated measures ANOVA (factors: time and conditions) and one-way ANOVAs for specific comparisons. Significant differences were observed between DA alone and DA + Fe^3+^ (≥1 μM Fe^3+^ accelerated DA degradation; *p* < 0.05).

Next, we investigated the effect of Fe^3+^ on DA degradation. The catechol group in DA (Figure 1A) readily coordinates with Fe^3+^ [71], and this complex formation influences degradation rates. Hedges et al. demonstrated that Fe^3+^ increases NM formation rates, with Fe^3+^ subsequently incorporating into NM deposits. This finding indicated that Fe^3+^ does not act as a catalyst in NM formation but instead becomes part of the final product [62]. Here, we explored whether Fe^3+^ also accelerated DA degradation. Fe^3+^ addition led to increased accumulation of insoluble brown particles corresponding to NM, confirmed by temporal UV absorbance increases at 650 nm (Figure 2A). Furthermore, Fe^3+^ exhibited concentration-dependent catalytic effects on the formation rates of phenolic organic compounds (likely DAC or DHI; Figure 2B) and AC (Figure 2C). These findings demonstrate that Fe^3+^ enhances NM formation and significantly accelerates DA degradation.

We then studied whether the presence of oxidants, reductants, and chelators influenced the catalytic effect of Fe^3+^ on DA degradation. The combined presence of Fe^3+^ and H_2_O_2_ significantly increased the formation rates of DAC, DHI, AC, and NM (Figure 2D) to a much greater extent than when either Fe^3+^ (Figure 2A) or H_2_O_2_ (Figure S5B) were present alone. This demonstrated that the co-presence of Fe^3+^ and pro-oxidants had a severe impact on free DA, accelerating its degradation. In contrast, the addition of a reductant such as TCEP completely inhibited the catalytic effect of Fe^3+^ on DA degradation. Only a slight increase was observed in the band corresponding to phenolic compounds (~290 nm) and the appearance of a broad band at ~580 nm, which could not be assigned (Figure 2E). These features were absent in the spectra of DA in the presence of TCEP alone (Figure S5E), suggesting that Fe^3+^, even in the presence of reductants, could still contribute to DA degradation, albeit minimally. Finally, we attempted to inhibit the catalytic effect of Fe^3+^ by adding a strong Fe^3+^ chelator, EDTA. Chelation of Fe^3+^ significantly reduced its catalytic activity on AC formation and decreased NM production. The temporal variation of the UV-Vis spectra of DA in the presence of EDTA (Figure 2F) closely resembled that of DA incubated alone (Figure S5A), indicating that chelating Fe^3+^ effectively neutralized its impact on DA degradation.

Our findings demonstrate that free Fe^3+^ cations significantly accelerate the degradation of DA and the formation of NM. The mechanism likely involves the reduction of Fe^3+^ to Fe^2+^, coupled with the oxidation of DA. However, this effect is effectively counteracted by the addition of chelating agents that sequester free Fe^3+^.

#### 2.1.6. On the Effect of Ca^2+^, Zn^2+^, and Al^3+^ on the Degradation of DA

In addition to Fe^3+^, other cations may also stimulate DA degradation. Although Ca^2+^ can coordinate with catechols [72], it cannot induce melanogenesis per se. However, it can do so in the presence of oxidants [62]. Al^3+^ binds to catechols and catalyzes the conversion of AC to DHI [73], whereas the co-presence of DA and Zn^2+^ has been shown to synergistically enhance cell death and DA depletion in the striatum [74]. To investigate whether Ca^2+^, Al^3+^, or Zn^2+^ could accelerate DA degradation, we examined their effects on the temporal UV-Vis spectral changes of DA. Unlike Fe^3+^, none of these cations influenced the temporal changes in the UV-Vis spectrum of DA when incubated alone (Figure S6A–C). Specifically, these cations exhibited no concentration-dependent catalytic effects on the formation rates of phenolic organic compounds (Figure S6D–F) or AC. Thus, our results indicate that other cations implicated in neurodegeneration, such as Ca^2+^, Al^3+^, or Zn^2+^, have a significantly lower capacity to induce DA degradation compared to Fe^3+^.

#### 2.1.7. Effect of pH and Buffer-Type on the Degradation of DA

We finally studied the effects of pH and buffer type on the degradation rate of DA. Temporal variations in the UV-Vis spectra of DA were recorded using a 10 mM phosphate buffer supplemented with 75 mM NaCl at pH 6.0, 6.5, and 7.4 (Figure S7A–C). The results demonstrate that both the degradation rate and the yield of DA oxidation products are strongly pH-dependent. At pH 7.4, the formation of polyphenols (as evidenced by the band at ~300 nm), AC (as indicated by the band at ~475 nm), and NM (evidenced by the band at ~650 nm and confirmed through visual inspection) was clearly observed. In contrast, NM formation was completely abolished at pH 6.0 and 6.5. At these pH values, only small amounts of polyphenols and AC were detected, and their concentrations were significantly lower at pH 6.0 than at pH 6.5.

We then studied the effect of buffer type on the Fe^3+^-catalyzed degradation rate of DA. Five buffers with varying chemical compositions and buffering capacities were tested: MES (pH 5.5–7.0), HEPES (pH 6.8–8.2), TRIS (pH 7.0–9.2), MOPS (pH 6.5–7.9), and phosphate (pH 5.8–8.0) (Figure S8). The data demonstrate that the chemical nature of the buffer significantly impacts the Fe^3+^-catalyzed degradation rate of DA. Among the buffers tested, phosphate exhibited the greatest capacity to promote the Fe^3+^-catalyzed degradation of DA, as well as the subsequent formation of AC and NM. This effect was concentration-dependent (Figure S3), indicating that both Fe^3+^ and phosphate can catalyze DA degradation. In contrast, the degradation of DA and formation of NM were markedly slower in TRIS compared to the other buffers. This can be attributed to the moderate ability of TRIS to chelate iron cations [75,76], whereas other buffers such as HEPES or MOPS have a much lower affinity for iron [77].

These results demonstrate that the degradation of DA and the formation of AC and NM are highly sensitive to small variations in pH. Furthermore, our findings confirm that buffer composition significantly influences the Fe^3+^-catalyzed degradation of DA and the formation of AC and NM, likely due to the iron-chelating properties of the buffer or its capacity to catalyze specific molecular pathways involved in DA oxidation.

### 2.2. IP6 Inhibits the Fe^3+^-Catalyzed Degradation of DA

Understanding the influence of environmental factors on DA degradation is crucial given its critical role in neurotransmission [64]. DA is synthesized in dopaminergic neurons through the sequential conversion of L-Phe to L-Tyr, which is further transformed into L-levodopa and finally into DA. Subsequently, DA is stored in SVs and released into the synaptic cleft, where it binds to DA receptors. Following release, DA may either be reabsorbed by DA transporters and repackaged into SVs or degraded via one of two enzymatic pathways: (i) the monoamine oxidase pathway, which converts DA into 3,4-dihydroxyphenylacetaldehyde (DOPAL) and then into 3,4-dihydroxyphenylacetic acid (DOPAC); and (ii) the catechol-*o*-methyltransferase pathway, which converts DA into 3-methoxytyramine. Both pathways ultimately converge to form homovanillic acid, a metabolite excreted in the urine [78].

Typical DA concentrations in the cytoplasm of the substantia nigra range between 2 and 5 μM [79]. These concentrations are essential for proper neurotransmission but are highly sensitive to dysregulated Fe^3+^ metabolism, which profoundly disrupts DA homeostasis. Excess of Fe^3+^ triggers multiple detrimental effects: it disrupts enzymatic functions (leading to imbalances in DA production), accelerates DA degradation via NM formation (Figure 2), increases the production of DOPAL and ROS, and impairs DA transporter activity, thereby reducing DA reuptake from the synaptic cleft [62,80,81]. These pathological processes are particularly significant in neurodegeneration, a condition that both arises from and exacerbates elevated Fe^3+^ levels in the brain [82,83,84]. Consequently, Fe^3+^-chelating pharmacological interventions could mitigate these effects and restore DA homeostasis.

Given the ability of IP6 to chelate Fe^3+^ (Figure 1B) [57,58] and its presence in neurons at μM concentrations [85], we propose that IP6 may act as a protective agent against Fe^3+^-induced DA degradation.

#### 2.2.1. IP6 Inhibits the Fe^3+^-Catalyzed Degradation of Free DA

Most DA is stored in SVs, whose lumen maintains a slightly acidic pH (5.5–6.0) [86]. This acidic environment is crucial for the proper function of vesicular transporters and the structural integrity of vesicle proteins. The low pH is maintained by vacuolar-type H^+^-ATPases, which actively pump protons into the vesicles. The resulting H^+^ gradient drives the exchange of H^+^ for DA via vesicular transporters, ensuring efficient DA storage [78]. Consequently, we studied whether IP6 could protect DA from Fe^3+^-catalyzed degradation at pH 6.0 by using MES buffer to simulate the vesicular pH.

**Figure 3:**
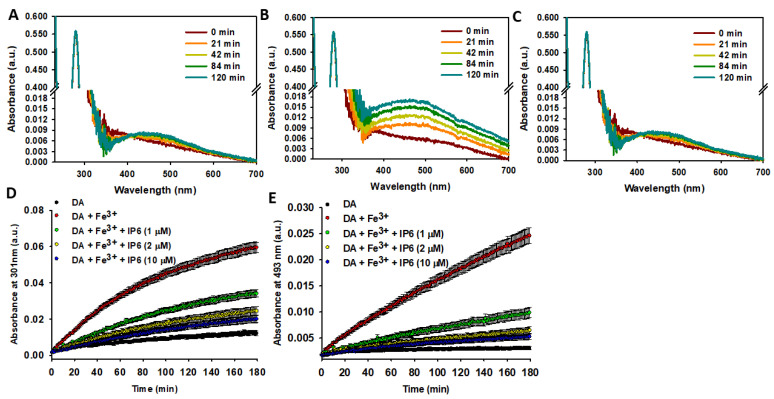
Effect of IP6 on Fe^3+^-catalyzed degradation of DA. (**A**–**C**) Temporal variation of UV-Vis spectra for a solution containing DA (100 μM) incubated at 37 °C in 20 mM MES buffer (pH 6.0) with 75 mM NaCl: (**A**) without Fe^3+^ nor IP6; (**B**) with Fe^3+^ (10 μM); (**C**) with Fe^3+^ (10 μM) and IP6 (1 μM). (**D**) Temporal variation of absorbance at 301 nm for a solution containing DA (100 μM) alone (black dots) or with Fe^3+^ (10 μM) and varying IP6 concentrations (0, 1, 2, and 10 μM). (**E**) Temporal variation of absorbance at 493 nm under the same conditions described in panel (**D**). Statistics: Data in panels (**D**,**E**) (*n* = 3) were analyzed using a two-way repeated measures ANOVA (factors: time and conditions) to assess the effect of IP6 on Fe^3+^-driven DA oxidation. Fe^3+^ significantly increased DA oxidation compared to DA alone (*p* < 0.05). IP6 (≥1 μM) significantly inhibited Fe^3+^-induced DA degradation (*p* < 0.05 vs. Fe^3+^ alone). Increasing IP6 from 1 μM to 2 or 10 μM further enhanced inhibition (*p* < 0.05).

Unlike the results obtained when DA was incubated in phosphate at pH 7.4 (Figure S3C), incubation in MES at pH 6.0 did not significantly alter its UV spectrum profile. This suggests that replacing phosphate with MES and/or decreasing the pH (from 7.4 to 6.0) effectively inhibits DA autoxidation (Figure 3A,D,E). The addition of Fe^3+^ (10 μM) stimulated the formation of polyphenols (indicated by the band appearing at ~301 nm), NM (evidenced by the increased absorbance at ~600 nm), and notably AC (indicated by the band at ~490 nm) (Figure 3B,D,E). Hence, Fe^3+^ is capable of catalyzing DA degradation even at pH 6.0. However, the catalytic effect of Fe^3+^ was completely neutralized when IP6 was added. In fact, after 120 min of incubation with Fe^3+^ and IP6, the UV-Vis spectrum of DA was nearly identical to that observed prior to incubation. Only a slight increase in the band at ~493 nm was detected, suggesting minimal formation of AC (Figure 3C). The IC_50_ of IP6 was ~1 μM, a concentration 10 times lower than that of Fe^3+^ (10 μM). Increasing the concentration of IP6 to 2 μM slightly enhanced its inhibitory effect, but further increases to 10 μM had a negligible impact (Figure 3D,E). This behavior can be attributed to the high affinity of IP6 for Fe^3+^ and its ability to chelate one to four Fe^3+^ ions under physiological conditions [87].

Consequently, our data reveal that IP6 exhibits remarkable protective properties against Fe^3+^-catalyzed DA oxidation.

#### 2.2.2. IP6 Inhibits the Fe^3+^-Catalyzed Degradation of Liposome-Encapsulated DA

**Figure 4:**
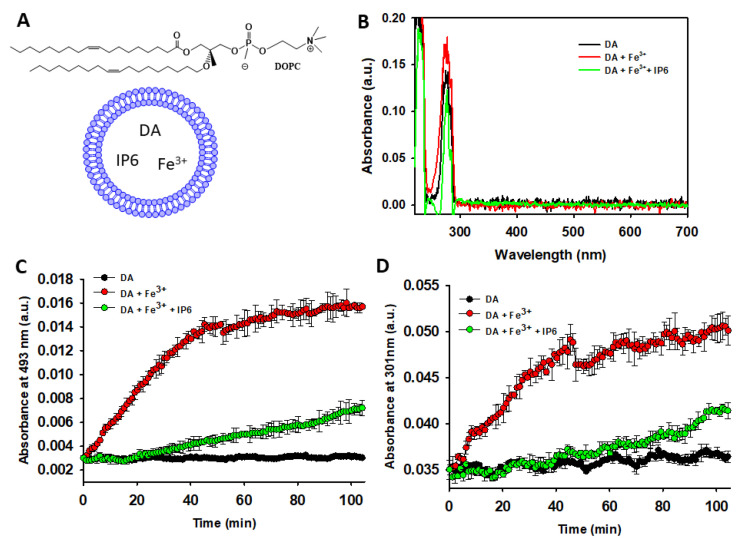
Studying the ability of IP6 to inhibit Fe^3+^-catalyzed DA degradation inside of SUVs. (**A**) Chemical structure of 1,2-dioleoyl-sn-glycero-3-phosphocholine (DOPC) and graphical representation of SUVs containing encapsulated DA, IP6, and Fe^3+^. Encapsulated DA and Fe^3+^ within the aqueous core remain solvated. IP6 is likely bound to cationic DOPC in the absence of Fe^3+^, while the soluble Fe^3+^-IP6 complex would predominate in Fe^3+^-containing conditions. Non-encapsulated molecules were removed via dialysis, ensuring DA, Fe^3+^, and IP6 remain isolated from the SUV outer hydrophilic layer. (**B**) UV-Vis spectra of disaggregated DOPC-SUVs (using 1% Triton X-100) assembled in 20 mM MES buffer (pH 6.0) with 75 mM NaCl and 1 mM DA (black), 1 mM DA and Fe^3+^ (10 μM) (red), or 1 mM DA, 10 μM Fe^3+^, and 50 μM IP6 (green). (**C**) Temporal variation of the absorbance at 493 nm of a solution containing DA (1 mM) in a 20 mM MES buffer (pH 6.0) that also contained 75 mM NaCl. The data were collected in the absence (black dots) and in the presence of Fe^3+^ (10 μM) with or without IP6 (50 μM). In this study, DA, Fe^3+^, and IP6 were encapsulated in DOPC-SUVs. (**D**) The same as described in panel C, but the absorbance was collected at 301 nm. Statistics: Data shown in panels (**C**,**D**) (*n* = 3) were analyzed via two-way repeated measures ANOVA (factors: time and conditions). DA and Fe^3+^ (encapsulated) vs. DA alone (encapsulated): Fe^3+^ significantly increased DA oxidation (*p* < 0.05). Fe^3+^ + DA + IP6 (encapsulated) vs. DA + Fe^3+^ (encapsulated): IP6 markedly reduced Fe^3+^-induced DA oxidation (*p* < 0.05). DA + Fe^3+^ + IP6 vs. control (DA alone): we did not observe significant difference in DA degradation.

Since intraneuronal DA is primarily encapsulated within SVs, we further investigated whether IP6 could also inhibit the degradation of DA encapsulated in small unilamellar vesicles (SUVs), which mimic SVs. The SUVs were assembled using the cationic lipid DOPC (Figure 4A) in a 20 mM MES buffer (pH 6.0) containing 1 mM DA. The encapsulation efficiency of DA into the DOPC-SUVs, as determined by the described protocol, was confirmed by analyzing UV-Vis spectra of Triton X-100-disrupted SUVs in the presence or absence of Fe^3+^ (10 μM) and/or IP6 (50 μM) (Figure 4B). The spectra showed no bands characteristic of polyphenols, AC, or NM formation, indicating that vesicle encapsulation combined with a low pH (~6.0) effectively protects DA from autoxidation.

Even incubation of DA-containing vesicles at 37 °C did not result in the formation of polyphenols or AC, as evidenced by the negligible changes in UV-Vis absorbance at 301 and 493 nm (Figure 4C,D). Consequently, vesicle encapsulation completely prevented DA from progressing to NM formation, as no blackish insoluble particles characteristic of NM (Figure S2) were observed in the incubated DA-containing vesicles. In contrast, UV-Vis data clearly demonstrate that the addition of Fe^3+^ to encapsulated DA stimulates its autoxidation, leading to the formation of AC and polyphenols. However, this Fe^3+^-induced oxidation was significantly inhibited by the presence of IP6 (Figure 4C,D).

Hence, these data demonstrate that vesicle encapsulation protects DA from autoxidation. However, autoxidation is enhanced when encapsulation occurs in the presence of free Fe^3+^. Notably, this metal-induced catalytic effect can be inhibited by encapsulated IP6. These findings suggest that IP6 could offer protection against DA autoxidation even within the lumen of SVs.

### 2.3. IP6 Inhibits the Fe^3+^-Catalyzed Degradation of AA and the Formation of ROS

In addition to DA, neurons contain other small molecules essential for proper neurotransmission and the maintenance of neuronal redox balance. One such molecule is AA (Figure 1B), which is highly concentrated in neurons and plays a crucial role in their function [65,66]. However, an excess of free Fe^3+^ stimulates AA degradation, leading to the concomitant production of ROS and disruption of normal neuronal function [88,89]. Given these effects, we investigated whether IP6 could also inhibit the Fe^3+^-catalyzed AA degradation and prevent ROS formation.

**Figure 5:**
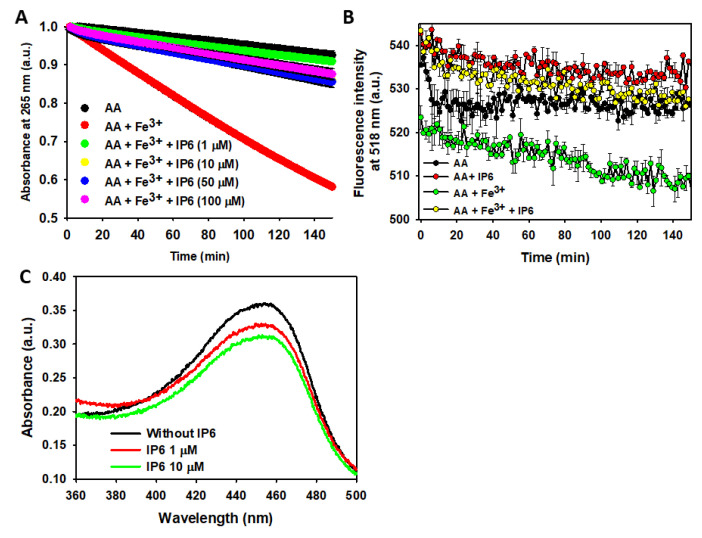
Effect of Fe^3+^ and IP6 on AA degradation and ROS formation/scavenging. (**A**) UV-Vis study of the time-dependent AA (70 μM) degradation at 25 °C, monitored by absorbance decay at 265 nm when AA was alone (black), in the presence of Fe^3+^ (2.5 μM) (red), in the presence of Fe^3+^ (2.5 μM) and IP6 (1 μM) (green), in the presence of Fe^3+^ (2.5 μM) and IP6 (10 μM) (yellow), in the presence of Fe^3+^ (2.5 μM) and IP6 (50 μM) (blue), and in the presence of Fe^3+^ (2.5 μM) and IP6 (100 μM) (purple). (**B**) Time-dependent ROS formation (via fluorescein (10 μM) fluorescence decay at λ_exc_ 490 nm) of a solution prepared in 10 mM sodium phosphate buffer containing 150 mM NaCl (pH 7.4) and (i) AA (70 μM) alone (black); (ii) AA (70 μM) and Fe^3+^ (2.5 μM) (green); (iii) AA (70 μM) and IP6 (50 μM) (red); and (iv) AA (70 μM), Fe^3+^ (2.5 μM), and IP6 (50 μM) (yellow). In both panels, the data points are the means from all the replicas, and the error bars represent the standard deviation from the different independent measurements. (**C**) UV–Vis spectra of the neocuproine-Cu^+^ complex formed from HO·-mediated salicylic acid hydroxylation in the absence (black) or in the presence of either 1 μM IP6 (red) or 10 μM IP6 (green). All the experiments were carried out in triplicate. Statistics: Kinetic data shown in panels (**A**,**B**) (*n* = 3) were analyzed by two-way repeated measures ANOVA (factors: time and conditions). The differences between the UV-Vis spectra of panel C were studied using the one-way ANOVA (with Tukey or Dunnett post hoc tests). Panel (**A**): Fe^3+^ significantly accelerated AA degradation vs. AA alone (*p* < 0.05). IP6 (≥1 μM) markedly inhibited Fe^3+^-catalyzed AA oxidation (*p* < 0.05 vs. Fe^3+^ alone). No dose-dependent improvement observed at higher IP6 concentrations (*p* > 0.05). Panel (**B**): Fe^3+^ increased ROS production (reflected by a faster fluorescein decay rate, *p* < 0.05 vs. AA alone). IP6 (50 μM) reversed Fe^3+^-induced ROS generation (*p* < 0.05 vs. Fe^3+^; no difference vs. AA alone). IP6 alone did not affect fluorescein decay (*p* > 0.05 vs. AA alone). Panel (**C**): IP6 (1–10 μM) significantly reduced HO· levels (*p* < 0.05 vs. control). No difference between 1 μM and 10 μM IP6 (*p* > 0.05), suggesting saturation at low doses.

We previously demonstrated that the degradation rate of AA is accelerated by increasing phosphate concentrations but slowed by elevated NaCl concentrations [90], likely due to the inhibitory effect of NaCl on the AA-O_2_ interaction [91]. To minimize non-catalyzed AA degradation, we analyzed its time-dependent decay in a 10 mM phosphate buffer (pH 7.4) containing 150 mM NaCl. Under these conditions, only ~8% of AA degraded after 150 min of incubation. However, adding a low concentration of Fe^3+^ (2.5 μM) markedly accelerated AA oxidation, with ~42% degraded within the same period. Strikingly, trace amounts of IP6 (i.e., ~1 μM) nearly abolished the pro-oxidant effect of Fe^3+^. Increasing the IP6 concentration (from 1 μM to 100 μM) did not further enhance inhibition, indicating that (i) IP6-chelated Fe^3+^ cannot catalyze AA oxidation; and (ii) an IP6:Fe^3+^ molar ratio of 1:2.5 is sufficient to sequester most free Fe^3+^ (Figure 5A). Thus, IP6 effectively protects neurons from the Fe^3+^-catalyzed degradation of AA.

The metal-catalyzed degradation of AA is not harmless to neurons, as it produces ROS [88,89]. ROS formation can be monitored using fluorescein, whose fluorescence emission decreases upon reaction with ROS [90,92]. Although the yield of ROS from the Fe^3+^-catalyzed AA degradation is lower compared to other metal cations (e.g., Cu^2+^) [90], Fe^3+^ induces a more rapid fluorescence decrease than is observed in its absence. Hence, Fe^3+^ directs part of the AA autoxidation pathway toward ROS formation. As expected, the addition of IP6 to the AA-Fe^3+^ reaction mixture completely prevented this fluorescence decay, demonstrating that IP6 can also indirectly reduce the neuronal ROS formation resulting from Fe^3+^-catalyzed reactions (Figure 5B).

Another intriguing question is whether IP6 can directly scavenge preformed ROS. To explore this, we assessed the ability of IP6 to neutralize HO· radicals generated via the CUPRAC assay. Adding 1 μM IP6 to the reaction mixture decreased the UV-Vis absorbance associated with neocuproine-Cu^+^ complex formation, indicating effective HO· scavenging and prevention of its reaction with the neocuproine-Cu^2+^ complex (Figure 5C). Increasing the IP6 concentration tenfold (10 μM) did not significantly enhance this effect compared to 1 μM IP6. This demonstrates that even tiny amounts of IP6 are sufficient to neutralize HO· radicals effectively.

Hence, our findings show that IP6 prevents Fe^3+^-catalyzed AA degradation, thereby reducing intraneuronal ROS formation. Additionally, IP6 exhibits direct scavenging potential against preformed ROS, such as HO· radicals.

### 2.4. Effect of IP6 on the Fibrillization of α-Synuclein

In addition to catalyzing the oxidation of DA and AA, Fe^3+^ promotes neurodegeneration through other molecular mechanisms. One key mechanism is its ability to stimulate the aggregation of proteins implicated in neurodegenerative disorders, such as tau [93] and Aβ_1–42_ peptides [94]. Elevated Fe^3+^ levels have been detected in neuronal protein aggregates [95], including Lewy bodies (LBs). LBs are primarily composed of αS, a small presynaptic and intrinsically disordered protein. These Fe^3+^-containing aggregates accumulate in the cytosol of dopaminergic neurons within the substantia nigra [96] and are a hallmark pathological feature of Parkinson’s disease.

Given the ability of IP6 to chelate Fe^3+^, we hypothesized that IP6 could inhibit Fe^3+^-induced protein aggregation, thus offering additional neuroprotection. To test this, pure monomeric αS was incubated under fibrillization-promoting conditions (i.e., at 37 °C in 20 mM phosphate buffer (pH 7.4) while shaking at 1000 rpm). Incubations were carried out in the absence or presence of Fe^3+^ and/or IP6. Aliquots were collected at different incubation times, diluted, and analyzed for amyloid fibril formation by measuring Tht fluorescence spectra.

**Figure 6:**
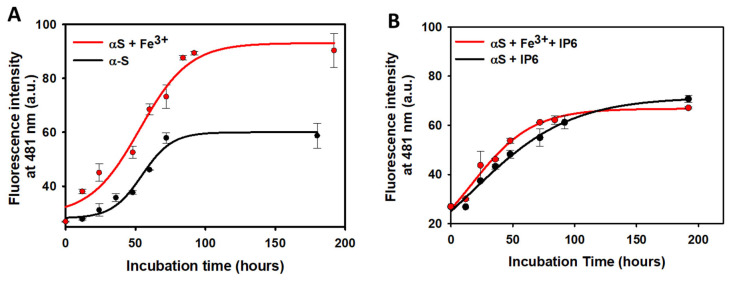
Effect of IP6 on the kinetics of αS amyloid fibril formation. (**A**) Time-dependent variation in fluorescence emission at 481 nm (λ_exc_ 440 nm) of solutions containing 120 μM monomeric αS prepared in 20 mM phosphate buffer (pH 7.4) with 150 mM NaCl, either without (black line) or with Fe^3+^ (2 μM) (red line). Solutions were incubated at 37 °C while shaking, and aliquots were taken at various incubation times and diluted to an αS concentration of 10 μM prior to fluorescence measurement. (**B**) Time-dependent variation in fluorescence emission at 481 nm (λ_exc_ 440 nm) of solutions containing 120 μM monomeric αS in 20 mM phosphate buffer (pH 7.4) with 150 mM NaCl and IP6 (20 μM), either without (black line) or with Fe^3+^ (2 μM) (red line). Solutions were incubated at 37 °C while shaking, and aliquots were taken at different incubation times and diluted up to an αS concentration of 10 μM before fluorescence measurement. Statistics: Data shown in the panels (*n* = 3) were analyzed using a two-way ANOVA (factors: Fe^3+^ and IP6) on aggregation endpoints (e.g., plateau Tht fluorescence intensity). Fe^3+^ effect without IP6: in the absence of IP6, Fe^3+^ significantly increased the final fibril yield compared to αS incubated without Fe^3+^ (*p* < 0.05). Fe^3+^ effect with IP6: in the presence of IP6, there was no significant difference between the +Fe^3+^ and −Fe^3+^ samples (*p* > 0.05), indicating that IP6 completely abolished the Fe^3+^-induced enhancement of αS aggregation. IP6 effect: the presence of IP6 resulted in a significantly lower Tht fluorescence plateau (indicating fewer amyloid fibrils formed) compared to the corresponding conditions without IP6 (*p* < 0.05, IP6 vs. no IP6).

As expected, incubation of monomeric αS resulted in the time-dependent formation of Tht-active aggregates, consistent with a nucleation-dependent mechanism. This process displayed a characteristic profile: an initial lag-phase (nucleation period), followed by a rapid fluorescence increase that culminated in a plateau (Figure 6A). The αS aggregates exhibited a linear and unbranched morphology typical of amyloid fibrils [97]. The addition of Fe^3+^ shortened the lag-phase compared to αS incubated alone, accelerating amyloid fibril formation. Furthermore, Fe^3+^ increased the fibril yield, as evidenced by a ~1.5-fold higher maximum Tht fluorescence intensity at the plateau phase relative to αS incubated without Fe^3+^.

The aggregation mechanism of monomeric αS was profoundly altered in the presence of IP6. IP6 eliminated the nucleation lag-phase, and the aggregation process followed an exponential growth profile until reaching maximum fluorescence intensity (Figure 6B). The maximum Tht fluorescence intensity at the plateau phase was significantly lower compared to αS incubated without IP6, suggesting that while IP6 accelerates αS aggregation, it reduces the overall formation of amyloid fibrils. Notably, Fe^3+^ did not alter the IP6-modified aggregation mechanism of αS (Figure 6), confirming that the chelating ability of IP6 neutralizes the stimulating effect of Fe^3+^ on the αS amyloid fibril formation.

During aggregation, the lag-phase represents the time required for monomers to assemble into soluble oligomers and their subsequent evolution into amyloid fibrils [98]. The ability of IP6 to accelerate the conversion of soluble oligomers into Tht-active amyloid fibrils could have significant neuroprotective implications. It is well-established that soluble oligomers, due to their hydrophobic nature, are the most toxic species formed during the aggregation, as they disrupt lipid membranes and induce cell death [99,100]. By eliminating the lag-phase, IP6 appears to rapidly redirect oligomers and protofibrils toward the formation of less harmful amyloid fibrils. This reduces the lifetime of toxic oligomers, thereby mitigating their potential to cause cellular damage.

While our data do not provide direct insights into the mechanism by which IP6 exerts its beneficial effects, several hypotheses can be proposed. One possibility is that IP6 binds to αS and catalyzes its clustering and fibril formation. Alternatively, IP6 may interact with soluble oligomers, promoting their rapid fibrillization. Further investigations are needed to fully elucidate how IP6 modifies the aggregation pathway of αS.

Our findings conclusively demonstrate that IP6 alters the aggregation mechanism of monomeric αS. Specifically, it destabilizes toxic soluble oligomers by accelerating their conversion into amyloid fibrils. Furthermore, IP6 completely neutralizes the Fe^3+^-induced enhancement of fibril yield. Collectively, these results suggest that IP6 could play a neuroprotective role by modulating protein aggregation pathways, thereby potentially mitigating neurodegeneration.

## 3. Discussion

Our study explores the neuroprotective role of IP6, particularly its capacity to mitigate Fe^3+^-catalyzed oxidative processes and protein aggregation pathways. Our findings indicate that IP6 not only protects DA and AA from Fe^3+^-induced degradation but also inhibits Fe^3+^-mediated aggregation of αS, a key protein implicated in the pathogenesis of Parkinson’s disease. Hence, we discuss these findings in the context of the current scientific knowledge on neurodegeneration, emphasizing the dual function of IP6 as both an iron chelator and an inhibitor of pathogenic protein aggregation.

### 3.1. IP6 Is a Protective Agent Against Fe^3+^-Catalyzed Degradation of DA

Free cytosolic DA plays an important role in neurotransmission, but it is also highly susceptible to oxidative degradation, particularly in the presence of Fe^3+^. We demonstrate that Fe^3+^ accelerates DA degradation by catalyzing its oxidation to AC and subsequent polymerization to NM. This mechanism is consistent with previous studies showing that Fe^3+^ promotes DA quinone formation and increases oxidative stress in dopaminergic neurons, which is a hallmark of neurodegenerative diseases [101].

IP6 significantly reduces the catalytic effect of Fe^3+^ on DA degradation, even at substoichiometric concentrations. This protective effect is likely due to the strong chelating ability of IP6 (*K_a_* > 10 M^−1^; [58]), which sequesters Fe^3+^ and prevents redox cycling. In fact, formation of the IP6-Fe^3+^ complex decreases the Fe^3+^/Fe^2+^ redox potential, making Fe^3+^ reduction more difficult. Consequently, IP6 stabilizes Fe^3+^ in a non-redox-active state, thereby preventing Fe^3+^-catalyzed oxidative damage [57]. Notably, inhibition of DA degradation by IP6 occurred at an IC_50_ of ~1 μM, highlighting its high efficiency. Our data suggest that IP6 blocks the pro-oxidant environment created by Fe^3+^, thereby maintaining DA integrity. This is particularly relevant given the vulnerability of neurons in the substantia nigra, where both DA and Fe^3+^ concentrations are physiologically high.

Moreover, this effect has broader implications for preventing early dopaminergic neurodegeneration. The interaction of Fe^3+^ with DA generates oxidation byproducts that amplify cellular damage and inflammatory responses in surrounding neuronal tissue. The ability of IP6 to neutralize these effects through Fe^3+^ chelation and oxidative damage prevention strengthens its potential as a therapeutic agent. Our results align with in vivo studies showing that IP6 protects cellular models of neurodegeneration from apoptosis [51] and exerts protective roles in Alzheimer’s and Parkinson’s diseases by (i) inhibiting ferroptosis; (ii) suppressing Ca^2+^-induced protein aggregation; (iii) exerting anti-neuroinflammatory effect by inhibiting microglial activation; (iv) reversing mitochondrial dysfunction; and (v) facilitating SV recycling and DA release [2].

### 3.2. Vesicle Encapsulation and the Role of IP6 in DA Stability

The study of encapsulated DA within SUVs mimicking SVs further underscores the protective potential of IP6. Due to its highly negative charge, IP_6_ can interact with cationic lipids of membranes, influencing both membrane properties and protein–lipid interactions [54]. Consequently, if encapsulated IP_6_ binds to lipids, it may hinder its ability to chelate Fe^3+^ and inhibit Fe^3+^-catalyzed DA oxidation. To investigate this interaction, we encapsulated DA, Fe^3+^, and IP6 within SUVs composed exclusively of the cationic lipid DOPC, a system designed to maximize IP_6_–lipid interactions.

Although vesicle encapsulation itself limits DA autoxidation, the addition of Fe^3+^ increases DA degradation even within this protective microenvironment. Remarkably, IP6 was able to counteract this Fe^3+^-induced destabilization, indicating that IP6 can exert its protective effect efficiently even under acidic intravesicular conditions (pH 6.0). This finding complements previous studies showing that IP6 interacts with Fe^3+^ at both physiological and subcellular pH levels [87]. Additionally, these results demonstrate that, despite the ability of IP6 to interact with cationic lipids, it still effectively protects DA from Fe^3+^-catalyzed oxidation within a vesicular environment, just as it does in aqueous solution. Consequently, this suggests that either IP_6_ does not bind significantly to the cationic lipids of the vesicles or, more likely, it binds while simultaneously sequestering free Fe^3+^ cations.

The dual role of IP6 as a vesicular DA stabilizer and a modulator of Fe^3+^-induced oxidative pathways further positions it as an innovative approach to safeguarding neurotransmitter systems. This function could be instrumental in addressing the progression of disorders characterized by vesicular dysfunction and oxidative stress, potentially bridging gaps in therapeutic approaches for diseases in which DA imbalance plays a role.

### 3.3. Inhibition of Fe^3+^-Catalyzed AA Degradation and ROS Formation

AA is another important molecule for normal neuronal function, as it acts as a primary antioxidant and a cofactor in neurotransmitter synthesis. However, it is highly susceptible to Fe^3+^-catalyzed degradation, which leads to ROS production and thereby exacerbates oxidative damage. Our data demonstrate that IP6 not only inhibits Fe^3+^-catalyzed AA degradation but also significantly reduces ROS formation, as evidenced by decreased fluorescein fluorescence.

Interestingly, the protective effect of IP6 on AA degradation was almost completely achieved at low concentrations (~1 μM), consistent with its high Fe^3+^-binding affinity. These findings are in agreement with previous data showing that IP6 prevents metal-catalyzed oxidation processes by forming stable complexes with Fe^3+^ [58]. Additionally, IP6 demonstrated the ability to directly scavenge HO· through mechanisms distinct from its Fe^3+^-chelating activity, suggesting a broad antioxidant role.

Since the Fe^3+^-catalyzed AA degradation contributes to neuronal oxidative stress and ROS formation, the dual role of IP6—as both a chelator and ROS scavenger—reinforces its potential as a neuroprotective agent. ROS not only trigger widespread cellular damage but also exacerbate neuroinflammatory cascades that further compromise neuronal viability. By attenuating these processes, IP6 emerges as a promising candidate for strategies aimed at reducing oxidative and inflammatory stressors in neurodegenerative contexts.

### 3.4. Modulation of α-Synuclein Aggregation by IP6

The aggregation of αS plays a key role in the pathology of Parkinson’s disease. This process begins when monomeric αS starts to self-associate into oligomeric intermediates. These oligomers can be on-pathway (leading to fibrils) or off-pathway (toxic, non-fibrillar aggregates) and can undergo self-association via hydrophobic and electrostatic interactions. During this process, known as the lag-phase, the associated oligomers act as nucleation seeds for further incorporation of αS monomers into growing protofibrils through a “templated” β-sheet stacking mechanism. Subsequently, protofibrils stack together to form mature amyloid fibrils [102]. Fe^3+^ not only accelerates the aggregation of αS but also increases the yield of amyloid fibrils, as demonstrated in this work. This finding is consistent with previous studies showing that Fe^3+^ facilitates αS aggregation and contributes to the formation of toxic Lewy bodies [93,96].

In the presence of IP6, the aggregation mechanism of monomeric αS was notably altered. IP6 abolished the nucleation lag-phase and directed the aggregation process toward the rapid formation of amyloid fibrils. Notably, the maximum Tht fluorescence intensity (an indicator of fibril quantity) was significantly lower in the presence of IP6, suggesting that it reduces the overall yield of fibrils. These results align with previous findings by Zhang et al., who reported that IP6 blocks the accumulation of cytosolic αS in SH-SY5Y cells treated with 6-hydroxydopamine [54]. We propose that IP6 accelerates fibril formation by directly binding to the cationic pocket in the 3D structure of αS protofibrils. IP6 carries nine negative charges at physiological pH, enabling it to bind to the 4–6 cationic charges within the cationic pocket of αS protofibrils (Figure S9). Once bound, the remaining negative charges on IP6 may facilitate monomer addition via electrostatic interactions with the K43-K45-H50 region, which would then be incorporated into the fibril core. Collectively, this mechanism would enhance monomer addition and fibril growth.

This shift in aggregation dynamics has critical neuroprotective implications. Soluble oligomers, rather than mature fibrils, are widely regarded as the most neurotoxic intermediates during αS aggregation. These oligomers interact with cellular membranes, disrupting lipids and triggering cell death [99,100]. By accelerating the conversion of toxic oligomers into less harmful fibrils, IP6 effectively reduces the lifetime and toxicity of these intermediates. This mechanism aligns with the emerging therapeutic strategies aimed at modulating aggregation pathways to minimize oligomer toxicity in neurodegenerative diseases.

### 3.5. Implications for Neurodegeneration Prevention

Our findings indicate that IP6 offers protection against several molecular mechanisms associated with neurodegeneration. By chelating Fe^3+^, IP6 prevents the oxidative degradation of DA and AA, both of which are critical for neuronal function. Furthermore, IP6 can modulate αS aggregation pathways, underscoring its potential to mitigate protein aggregation-driven toxicity.

The relevance of these findings is amplified in the context of neurodegenerative diseases such as Parkinson’s and Alzheimer’s, where dysregulated iron metabolism, oxidative stress, and toxic protein aggregates converge. Elevated Fe^3+^ levels in the substantia nigra and hippocampus are strongly associated with neuronal death and disease progression [59,83]. Therapeutic strategies targeting iron dysregulation are promising; however, the high specificity and low toxicity of IP6 towards Fe^3+^ offer a distinct advantage over traditional and synthetic iron chelators.

In addition, the interplay between oxidative stress and protein aggregation constitutes a vicious cycle in neurodegeneration. The ability of IP6 to intervene in both pathways highlights its potential as a multi-target therapeutic agent. These dual functions could serve as a foundation for novel drug development strategies aimed at mitigating multiple hallmarks of neurodegeneration.

### 3.6. Limitations of the Study, Clinical Implications, and Future Directions

Our study clearly demonstrates that IP6 possesses neuroprotective properties. However, our findings may not be fully replicable in the complex biochemical environment of the human brain, as they have certain limitations. This is because our in vitro measurements were conducted under non-physiological conditions, using buffer systems that do not fully replicate the intricate biochemical environment of living cells (e.g., using MES buffer at pH 6.0). Additionally, our experiments were performed in a cell-free system, which does not account for the presence of metal-regulating proteins or intracellular processes such as enzymatic metabolism or cellular uptake mechanisms that could influence DA stability and aggregation pathways. As a result, the direct comparability of our results with in vivo conditions is limited. Furthermore, we acknowledge that conclusions drawn from our experiments are only valid for **extracellular DA**, as the redox environment, metal ion availability, and enzymatic degradation mechanisms inside neurons could significantly alter DA behavior. Therefore, future studies should focus on validating the neuroprotective effects of IP6 in cellular and animal models, such as dopaminergic neurons exposed to Fe^3+^ or iron-overload mouse models of neurodegeneration. Additional studies should also explore the kinetics of IP6-mediated protection against DA and AA oxidation under cytoplasmatic-like or vesicular-like conditions. These investigations could be extended to other neurotoxic metal cations (e.g., Mn^2+^ or Cu^2+^) to determine whether the protective role of IP6 is metal-specific. Although we have suggested a possible molecular mechanism, the precise mechanisms underlying the effect of IP6 on αS aggregation remain unclear. Further studies are required to elucidate whether IP6 directly interacts with αS monomers, oligomers, or fibrils. Additional computational modeling approaches could provide valuable insights into these interactions, identifying key binding motifs and energy landscapes involved in the modulation of aggregation.

Finally, we are also aware that the neurotherapeutic potential of IP6 in clinical settings depends on its bioavailability, its ability to cross the blood–brain barrier, and its dose-dependent side effects. However, the latter appears to be a non-limiting factor, as mild side effects (e.g., nausea, headache, and insomnia) have only been reported at very high doses (>12 g/day in humans) [103]. In fact, IP6 is classified as “Generally Recognized As Safe (GRAS)” by the FDA, especially when consumed as part of a balanced diet supplemented by adequate mineral intake [104]. Most people consuming a Western or Mediterranean diet ingest between 1 and 2 g of IP6 per day without experiencing any side effect [104]. In addition, no significant IP6-drug interactions have been reported that could hamper its therapeutic use [105]. On the other hand, the dose–effect relationship in neurodegeneration is not well understood. However, it has been shown that an IP6 intake of 0.65 g/day is positively associated with improved cognitive function in older adults [48]. Furthermore, 30–100 µM IP6 reduces apoptosis and caspase-3 activity in Parkinson’s disease cell lines [51], and a 2% phytate diet reduces lipid peroxidation markers (e.g., malondialdehyde) and enhances mitochondrial function in Alzheimer’s disease [51,104]. Thus, it appears that moderate dietary intake (~1–2 g/day) may be sufficient for neuroprotection.

In any case, the clinical implications of IP6 might extend beyond its neuroprotective mechanisms, offering promising avenues for therapeutic development. Preclinical evidence supports the use of IP6 as a candidate for adjunctive therapy in neurodegenerative diseases, where its iron-chelating and anti-inflammatory properties could mitigate amyloid-beta toxicity and dopaminergic neuron loss. Furthermore, its synergy with chemotherapeutic agents—protecting neurons while enhancing cancer cell cytotoxicity—suggests potential applications in preventing chemotherapy-induced neuropathy. Emerging human data, such as from the ongoing PHYND trial investigating IP6 supplementation in diabetic cognitive impairment, in which we have participated [106], highlight its translational relevance.

## 4. Materials and Methods

### 4.1. Materials

1,2-dioleoyl-sn-glycero-3-phosphocholine (DOPC) was purchased from Avanti Polar Lipids (Alabaster, AL, USA). All the other chemicals and reagents used in this study were analytical grade and they were purchased either from Merck (Darmstadt, Germany) or from Fisher Scientific (Pittsburgh, PA, USA). All of them were used as received without further purification. All solutions used in this study were prepared using milli-Q water.

### 4.2. UV-Vis Spectroscopy Study of the Degradation of DA

A Shimadzu UV-2401-PC double beam spectrophotometer (Shimadzu Europa GmbH, Duisburg, Germany) was used to study the oxidation of DA under different experimental conditions. All the studies were carried out at 37 °C using a 1 cm quart cell. The UV-Vis spectra were collected from 220 to 900 nm at 5-min interval over a period of 2 h. In addition, under certain experimental conditions, kinetic studies were carried out at a fixed wavelength, which was either 301 or 493 nm.

To study the effect of DA concentration on its degradation rate, the UV-Vis spectra of solutions containing 30, 50, 100, 500, or 1000 μM DA concentrations were collected in 10 mM of phosphate (pH 7.4) containing 75 mM NaCl.

To study the effect of sodium phosphate concentration on the stability of DA, we acquired the UV-Vis spectra of solutions containing 100 μM DA prepared in mili-Q water; in 1 mM sodium phosphate; in 10 mM sodium phosphate; in 100 mM sodium phosphate; and in 500 mM sodium phosphate. The pH of all the solutions was 7.4, and all of them contained 75 mM NaCl.

To study the effect of NaCl concentration on the stability of DA, we acquired the UV-Vis spectra of solutions containing 100 μM DA prepared in 10 mM phosphate buffers (pH 7.4) that were prepared in the absence or in the presence of NaCl at 20, 75, or 300 mM concentrations.

To study the effect of the presence of oxidants or reductants on the stability of DA, we acquired the UV-Vis spectra of solutions containing 100 μM DA prepared in a 10 mM phosphate buffer (pH 7.4) containing 75 mM NaCl. The solutions were prepared in the presence of (i) H_2_O_2_ (50 μM); (ii) KIO_3_ (50 μM); (iii) AA (50 μM); or (iv) tris(2-carboxyethyl)phosphine (TCEP) (50 μM).

To study the effect of Fe^3+^ on the stability of DA, we collected the UV-Vis spectra of solutions containing 100 μM DA prepared in 10 mM phosphate buffer (pH 7.4) containing 75 mM NaCl. The solutions were prepared in the presence of (i) FeCl_3_ (5 μM); (ii) FeCl_3_ (5 μM) and H_2_O_2_ (50 μM); (iii) FeCl_3_ (5 μM) and TCEP (50 μM); and (iv) FeCl_3_ (5 μM) and EDTA (50 μM). In addition, we acquired the temporal variation of the absorbance at 301 and at 493 nm of the above-mentioned DA solution (100 μM) in the presence of Fe^3+^ at 1, 2, 5, and 10 μM concentrations. The solutions containing Fe^3+^ were supplied with glycine (8 mM) to slow down the precipitation of insoluble hydroxyls [90,107].

To study the effect of Ca^2+^, Al^3+^, and Zn^2+^ on the degradation rate of DA, we collected the UV-Vis spectra of solutions containing 100 μM DA prepared in 10 mM phosphate buffer (pH 7.4) containing 75 mM NaCl. The solutions were prepared in the presence of (i) CaCl_2_ (500 μM); (ii) ZnCl_2_ (50 μM); and (iii) AlCl_3_ (20 μM). In addition, we acquired the temporal variation of the absorbance at 301 and at 493 nm of the above-mentioned DA solution (100 μM) in the presence absence and in the presence of: (i) CaCl_2_ at 50, 300, and 500 μM concentrations; (ii) ZnCl_2_ at 2, 10, 20, and 50 μM concentrations; and (iii) AlCl_3_ at 2, 5, 10, and 20 μM concentrations.

To study the effect of pH on the DA degradation rate, we recorded the UV-Vis spectra of solutions containing 100 μM DA prepared in 10 mM phosphate buffers containing 75 mM NaCl. Different phosphate buffers were prepared. One at pH 6.0, another at pH 6.5, and the third one at pH 7.4.

To study the effect of the buffer type on the Fe^3+^-catalyzed degradation rate of DA, we recorded the UV-Vis spectra of solutions containing DA (1 mM) and Fe^3+^ (10 μM) prepared in different buffers containing 75 mM NaCl. The pH of the buffers used in these studies was set at 7.4 and they were as follows: (i) 20 mM MOPS; (ii) 20 mM TRIS; (iii) 20 mM MES; (iv) 20 mM; and (v) 20 mM phosphate. The solutions were supplied with glycine (8 mM) to slow down the precipitation of insoluble Fe^3+^ hydroxyls [90,107].

To study the effect of IP6 on the Fe^3+^-catalyzed degradation rate of DA, we collected the UV-Vis spectra of solutions containing 100 μM DA prepared in 20 mM MES buffer (pH 6.0) containing 75 mM NaCl. The spectra were collected in the absence and in the presence of Fe^3+^ (10 μM), but also in the presence of Fe^3+^ (10 μM) and IP6 (1 μM). In addition, we acquired the temporal variation of the absorbance at 301 and at 493 nm of the above-mentioned DA solution (100 μM) in the presence of Fe^3+^ (10 μM) with or without IP6 at different concentrations (i.e., 1, 2, and 10 μM). The solutions containing Fe^3+^ were supplied with glycine (8 mM) to slow down the precipitation of insoluble hydroxyls [90,107].

### 4.3. Preparation and UV-Vis Spectroscopy Study of DA-Encapsulated DOPC Vesicles

The DOPC vesicles were always freshly prepared just before performing the experiments. Aliquots from the commercial stock solution of DOPC (25 mg/mL in CHCl_3_) were diluted in CHCl_3_ up to 1 mM concentration to a final volume of 2 mL. Later on, the CHCl_3_ was removed using a rotary evaporator that worked under reduced pressure (290 mbar) and at 30 °C. The obtained lipid films were then exposed to N_2_ gas during 10 min to ensure the complete dryness, and afterwards, they were hydrated for 30 min with 2 mL of a degassed 20 mM MES buffer (pH 6.0) also containing 75 mM NaCl and DA (1 mM). The hydration process was additionally carried out using the same buffer but also containing FeCl_3_ (10 μM) or FeCl_3_ (10 μM) and IP6 (50 μM) in the presence of glycine (8 mM). In all cases, the lipid hydration was carried out in ice to prevent DA oxidation. The resulting solutions were used to prepare multilamellar vesicles (MLVs) by vortexing the lipid suspensions for 10 min. The MLVs were then used to prepare small unilamellar vesicles (SUVs) through 5 cycles of freezing (at −20 °C) and thawing (at room temperature). Then, they were extruded 15 times through a 50 nm-pore-size polycarbonate filter using the Avanti Polar Lipids (Alabaster, AL, USA) mini-extruder.

Afterwards, the 2 mL of DOPC-SUVs solution was dialyzed at 4 °C against 2 L of 20 mM MES buffer (pH 6.0) using a 1 KDa dialysis cassette. The dialysis was carried out at 4 °C in three different steps (using 650 mL of buffer in each step). We recorded the UV-spectrum of the buffer used in the last dialysis step to ensure that all the non-encapsulated DA was removed from the solution containing the DOPC-SUVs.

The presence of DA in the lumen of the DOPC-SUVs was assessed through the vesicle disaggregation (by adding 1% Triton X-100) and the subsequent measurement of the UV-spectra of the obtained solutions.

The degradation rate of the encapsulated DA in the absence and in the presence of Fe^3+^ and/or IP6 was monitored over time at 37 °C using UV-Vis spectroscopy. We simultaneously collected the absorbance increase at 301 nm (characteristic of the phenolic degradation byproducts [67,69]) and at 493 nm (typical of AC formation [67]). The measurements were performed in triplicate using 100 μM DOPC-SUVs solutions.

### 4.4. Ascorbic Acid (AA) Oxidation Rate

The degradation rate of AA (70 μM) was followed using a UV spectrometer by measuring the temporal absorbance variation at 265 nm for 150 min. The studies were carried out at 25 °C using a 1 cm quart cell and a Shimadzu UV-2401-PC double beam spectrophotometer (Shimadzu Europa GmbH, Duisburg, Germany). The measurements were carried out in a 10 mM sodium phosphate buffer containing 150 mM NaCl (pH 7.4), which was also used as reference. The degradation rate of AA was recorded in the absence or in the presence of Fe^3+^ (2.5 μM) with or without the presence of IP6 at different concentrations (i.e., 1, 10, 50, and 100 μM). The reaction mixtures that contained Fe^3+^ included glycine (8 mM) to slow down the precipitation of insoluble hydroxyls when adding Fe^3+^ to the phosphate buffer [90,107]. Control experiments were carried out at different concentrations of IP6 in the absence of Fe^3+^. All the experiments were carried out in triplicate to ensure reproducibility.

### 4.5. Study of the Total Free Radical Formation from Fe^3+^-Catalyzed AA Degradation

The formation of total reactive oxygen species (ROS) was indirectly studied from the temporal fluorescence emission intensity decrease of fluorescein, which was measured using a *λ*_*em*_ = 518 nm and at *λ*_*exc*_ = 490 nm [92]. A fluorescein stock solution (2 mM) was prepared in a 10 mM sodium phosphate buffer containing 150 mM NaCl (pH 7.4) and glycine (8 mM) added to a final concentration of 10 μM to solutions containing AA (70 μM) alone or in the presence of 2.5 μM Fe^3+^ with or without IP6 (1 μM). The temporal variation in the fluorescence signal was followed over 150 min. All the experiments were run in triplicate at 25 °C using a Varian Cary Eclipse fluorescence spectrophotometer (Walnut Creek, CA, USA) and quartz cells of 1 cm path length.

### 4.6. Scavenging Capacity of IP6 Against HO Radical

The cupric reducing antioxidant capacity (CUPRAC) method was applied to analyze the ability of IP6 to scavenge the HO· radical [108]. The CUPRAC method involves the formation of HO· as a result of the reaction between Fe^2+^ and H_2_O_2_. After this reaction is completed, the latter is degraded using catalase to avoid chemical interferences. HO· can hydroxylate salicylic acid, which further reduces neocuproine-Cu^2+^ to neocuproine-Cu^+^ (λ_abs_max_ 450 nm). If IP6 reacts with HO·, this would avoid the hydroxylation of salicylic acid and the formation of the UV-active neocuproine-Cu^+^ complex.

The day we performed the assay, different stock solutions were prepared and stored at 5 °C until used: 3000 U of bovine catalase, 10 mM of ammonium acetate, 10 mM of CuCl_2_, 7.5 mM of neocuproine, 10 mM of salicylic acid, 20 mM of a FeCl_2_ solution prepared in 1 M HCl, 20 mM of EDTA, and 10 mM of H_2_O_2_. All of these solutions were prepared in milli-Q water except the neocuproine, which was dissolved in ethanol, and the catalase, which was prepared in 10 mM phosphate buffer (pH 7.4).

Three different sets of experiments were prepared: (i) one of them did not include IP6; (ii) one included 1 µM IP6; (iii) and the other included 10 µM IP6. In addition, all the reaction mixtures contained salicylic acid (0.5 mM), Fe^2+^ (0.5 mM), EDTA (0.5 mM), and H_2_O_2_ (0.5 mM). Then, samples were incubated at 37 °C and shaken at 500 rpm for 10 min. Afterwards, catalase was added to a total amount of 7.5 U to eliminate the excess H_2_O_2_. The mixture was incubated at room temperature for 30 min, and meanwhile, a new reaction mixture was prepared containing 1 mM Cu^2+^, 0.75 mM neocuproine, 0.2 M ammonium acetate, and 100 µL of the reaction mixture. The samples were measured by UV-visible spectroscopy using as a blank the 100 µL of Milli-Q water instead of the reaction mixture. All the experiments were performed in triplicate to ensure reproducibility.

### 4.7. Fibril Formation from Human α-Synuclein (αS)

Recombinant human αS was produced and purified as we described before in many manuscripts published by our research group [90,97,109,110,111]. To obtain pure monomeric αS, the recombinant αS was further purified using size exclusion chromatography and a Superdex-75 HR 10/300 column (Cytiva, Marlborough, MA, USA), as carried out before [97]. Afterwards, monomeric αS (120 μM) was incubated under conditions that are well known to induce its fibrillization (i.e., at 37 °C in a 20 mM phosphate buffer (pH 7.4) containing 150 mM NaCl while shaking at 1000 rpm). The incubations were carried out on solutions containing the following: (i) αS alone; (ii) αS in the presence of Fe^3+^ (2 μM); (iii) αS in the presence of IP6 (20 μM); and (iv) αS in the presence of Fe^3+^ (2 μM) and IP6 (20 μM). The solutions containing Fe^3+^ were supplied with glycine (8 mM). Aliquots from each reaction mixture were taken at different incubation times and diluted up to a 10 μM αS concentration. Then, the resulting solutions were supplied with 50 μM of thioflavin T (Tht), used as fluorescent probe to monitor amyloid fibril formation [112], and the corresponding fluorescent spectra were collected between 490 and 600 nm using a *λ*_*exc*_ = 440 nm. The scan speed was 200 nm/min with an excitation and emission slit of 2.5 nm, while 5 scans were accumulated. For each reaction mixture, the fluorescent emission intensity obtained at 481 nm was plotted against the incubation time. Fluorescent measurements were carried out at 37 °C on a Cary Eclipse fluorescence spectrophotometer (Walnut Creek, CA, USA) equipped with a Peltier temperature-controlled cell holder.

### 4.8. Statistical Analysis

All experiments in this study were performed in three independent replicates, and data are presented as means ± standard error unless otherwise specified. Statistical analyses were carried out using IBM SPSS Statistics v23 (IBM Corp., Armonk, NY, USA). For time-course experiments, two-way repeated measures analysis of variance (ANOVA) was used to evaluate the effects of treatment and time, followed by Bonferroni post hoc tests when appropriate. For comparisons among multiple groups at a single time point, one-way ANOVA with Bonferroni post hoc multiple comparisons was applied. A *p*-value of less than 0.05 was considered statistically significant. Specific statistical tests and comparisons are described in the corresponding figure legends.

## Data Availability

The data presented in this study are available on reasonable request from the corresponding author.

## Supplementary Materials

The following supporting information can be downloaded at https://www.mdpi.com/article/10.3390/ijms26104799/s1.
