# Supplementary material for: On the Potential Role of Phytate Against Neurodegeneration: It Protects Against Fe3+-Catalyzed Degradation of Dopamine and Ascorbate and Against Fe3+-Induced Protein Aggregation
Source: Int J Mol Sci. 2025 May 16;26(10):4799. doi: 10.3390/ijms26104799 (PMC12112605; doi:10.3390/ijms26104799)
Supplement: Supplementary file 1 [file ijms-26-04799-s001.zip › ijms-3576252-supplementary.pdf]

Supplementary Information for

On the potential role of phytate against neurodegeneration: it protects against Fe3+-catalyzed degradation of dopamine and ascorbate, and against Fe3+-induced protein aggregation

Samantha Rebeca Godoy, Pilar Sanchis, Juan Frau, Bartolomé Vilanova and Miquel Adrover*

Institut Universitari d'Investigació en Ciències de la Salut (IUNICS). Institut d'Investigació Sanitària Illes Balears (IdISBa). Departament de Química, Universitat de les Illes Balears, Ctra. Valldemossa km 7.5, E-07122 Palma de Mallorca, Spain.

*Correspondence to: Miquel Adrover, University of Balearic Islands.

Phone: +34 971 173491, Fax +34 971 173426,

e-mail: miquel.adrover@uib.es

2.-SUPPLEMENTARY FIGURES

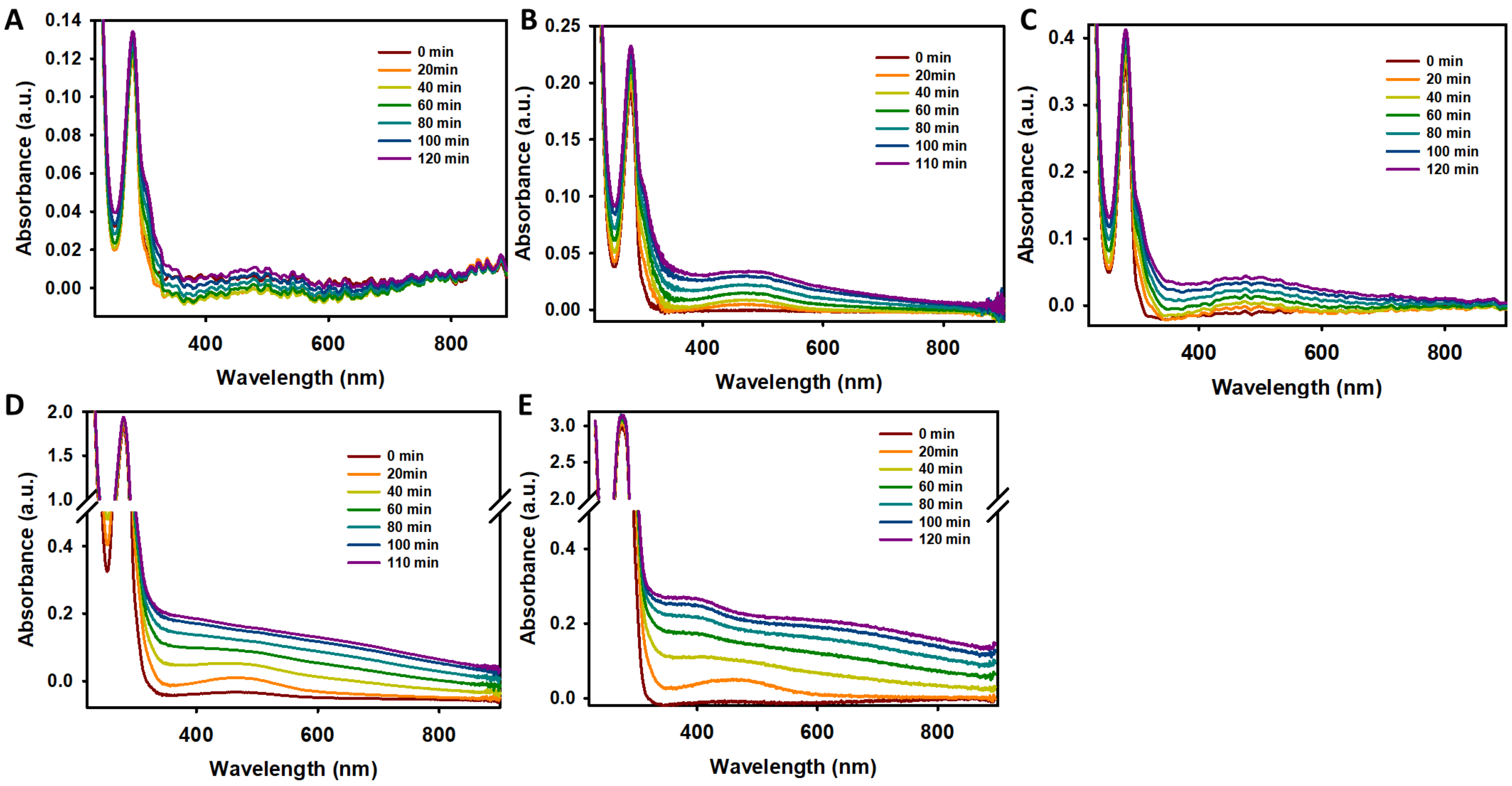

Figure S1. Effect of DA concentration on its degradation mechanism and rate. Temporal variation of the UV-Vis absorbance spectra of solutions containing DA at 30 µM (**A**), 50 µM (**B**), 100 µM (**C**), 500 µM (**D**) or 1 mM (**E**) concentrations. All solutions were prepared using a 10 mM phosphate (pH 7.4) containing 75 mM NaCl. All the spectra were recorded at 37°C.

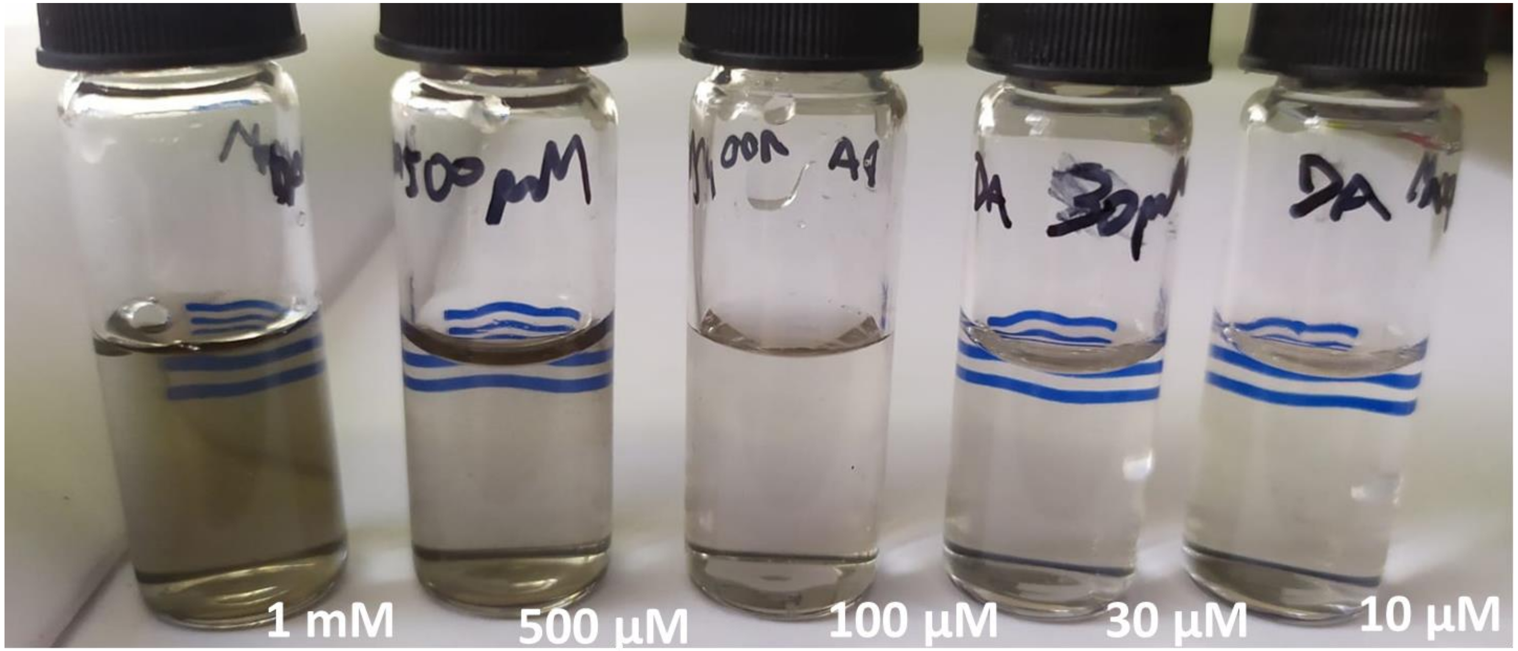

Figure S2. Formation of NM from solutions containing DA at different concentrations. Images of different vials containing DA solutions at different concentrations (i.e. 10 µM, 30 µM, 100 µM, 500 µM and 1mM). Before taking the pictures, the solutions were incubated in a 10 mM phosphate buffer (pH 7.4) containing 75 mM NaCl at 37°C during 2 hours. The black small insoluble particles formed after incubation were attributed to the formation of NM.

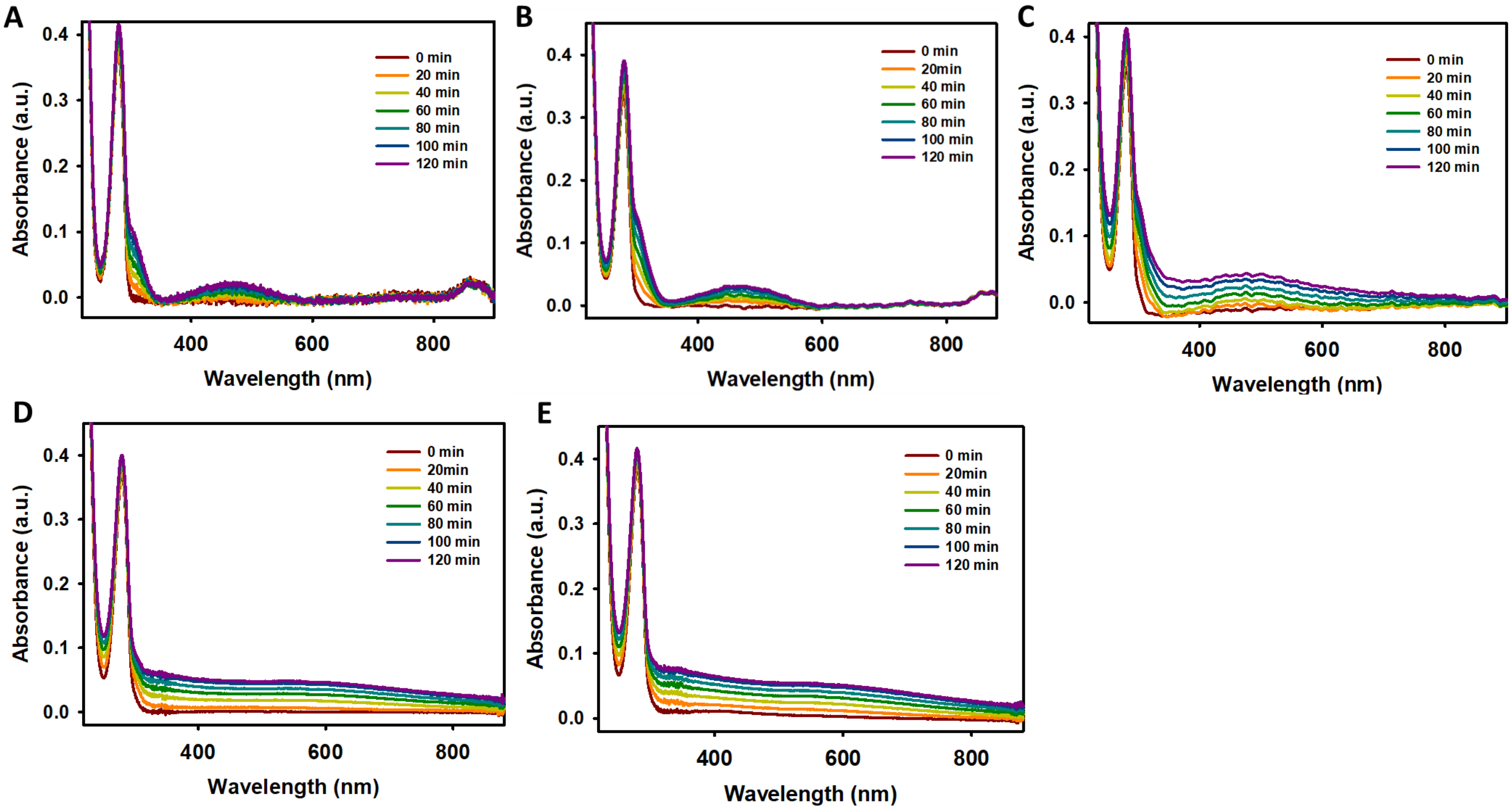

Figure S3. Effect of phosphate concentration on the degradation of DA. Temporal variation of the UV-Vis absorbance spectra of solutions containing DA at 100 µM which were prepared in: (**A**) a non-buffered aqueous solution; (**B**) a 1 mM phosphate buffer (pH 7.4) containing 75 mM NaCl; (**C**) a 10 mM phosphate buffer (pH 7.4) containing 75 mM NaCl; (**D**) a 100 mM phosphate buffer (pH 7.4) containing 75 mM NaCl; (**E**) a 500 mM phosphate buffer (pH 7.4) containing 75 mM NaCl. All the spectra were recorded at 37ºC.

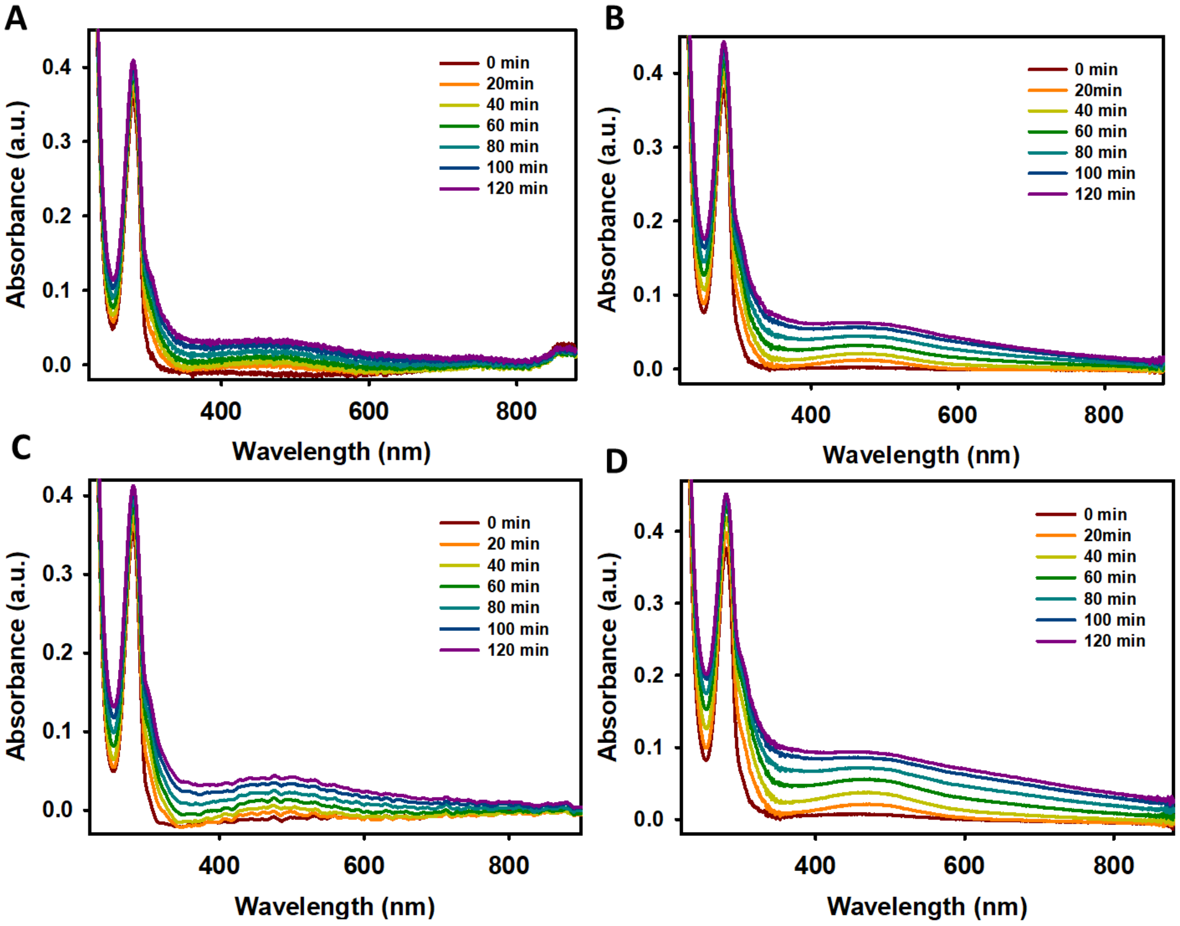

Figure S4. Effect of NaCl concentration on the degradation of DA. Temporal variation of the UV-Vis absorbance spectra of solutions containing DA at 100 µM which were prepared in a 10 mM phosphate (pH 7.4) in the absence (**A**) and in the presence of 20 mM (**B**), 75 mM (**C**) or 300 mM (**D**) NaCl. All the spectra were recorded at 37°C.

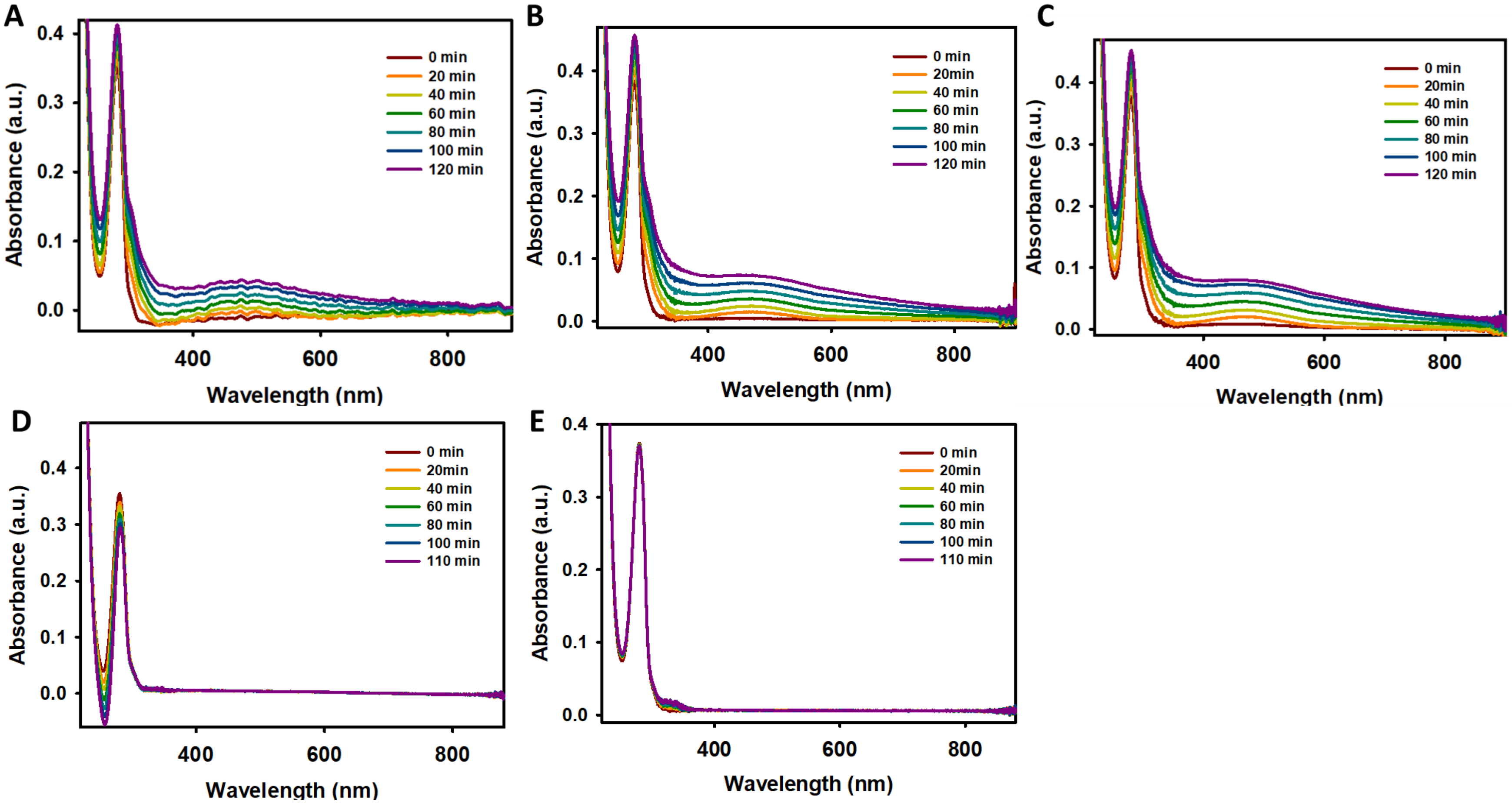

Figure S5. Studying the effect of the presence of oxidants and reductants on the degradation of DA. Temporal variation of the UV-Vis absorbance spectra of solutions containing DA at 100 µM in the absence (**A**), and in the presence of: 50 µM of H_2O_2 (**B**); 50 µM of KIO_3 (**C**); 50 µM of ascorbic acid (AA) (**D**); and 50 µM of tris(2-carboxyethyl)phosphine (TCEP) (**E**). All solutions were prepared using a 10 mM phosphate (pH 7.4) containing 75 mM NaCl and all the spectra were recorded at 37ºC.

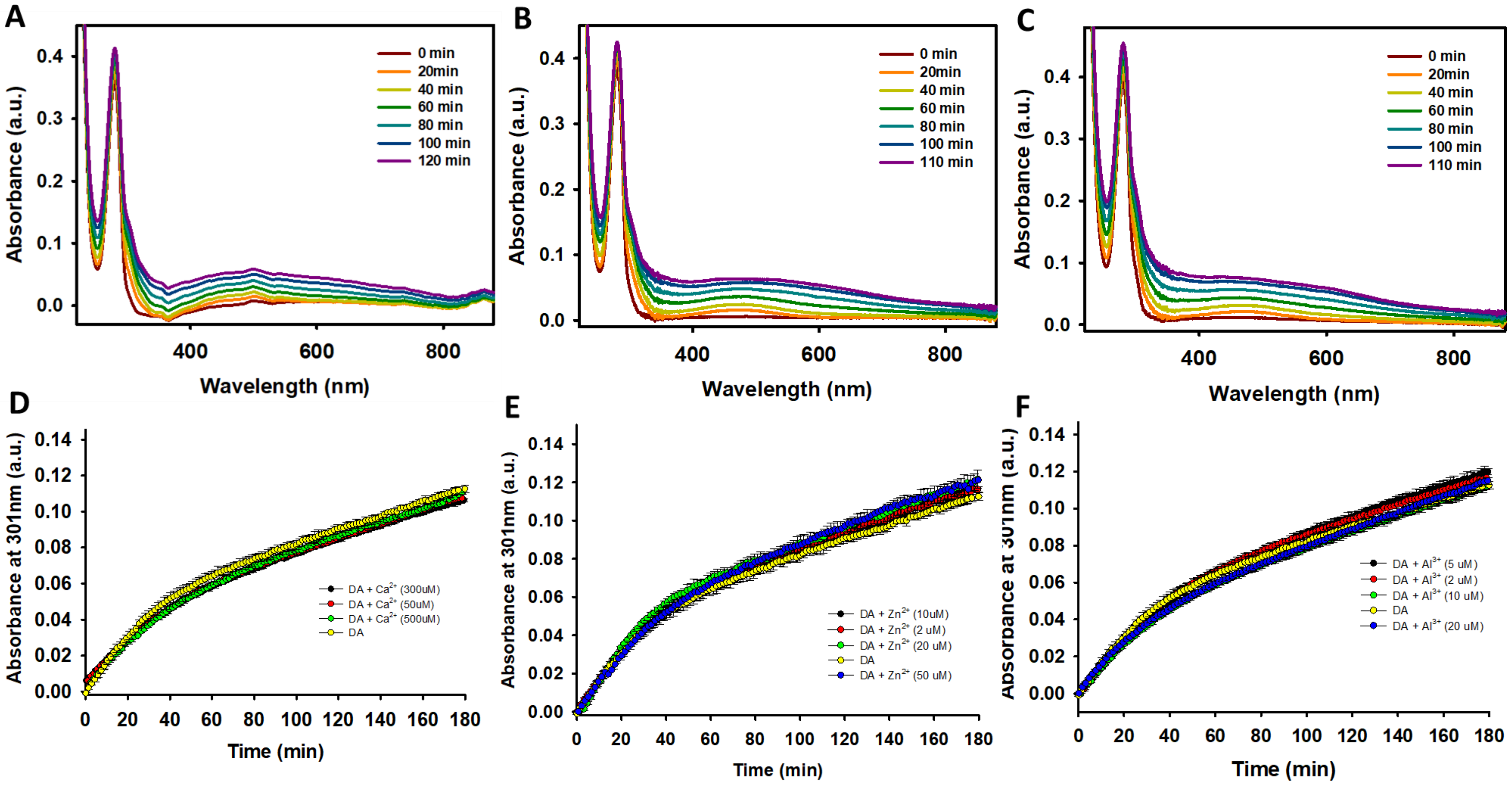

Figure S6. Studying the effect of Ca^{2+}, Al^{3+} and Zn^{2+} on the degradation of DA. (**A**) Temporal variation of the UV-Vis spectra of a solution containing DA (100 µM) in the presence of Ca^{2+} (500 µM). (**B**) Temporal variation of the UV-Vis spectra of a solution containing DA (100 µM) in the presence of Zn^{2+} (50 µM). (**C**) Temporal variation of the UV-Vis spectra of a solution containing DA (100 µM) in the presence of Al^{3+} (20 µM). (**D-F**) Temporal variation of the absorbance at 301 nm of a solution containing DA (100 µM) in the absence (*yellow dots*) and in the presence of different concentrations of Ca^{2+} (**D**), Zn^{2+} (**E**) or Al^{3+} (**F**). All solutions were prepared using a 10 mM phosphate (pH 7.4) containing 75 mM NaCl and all the spectra were recorded at 37°C.

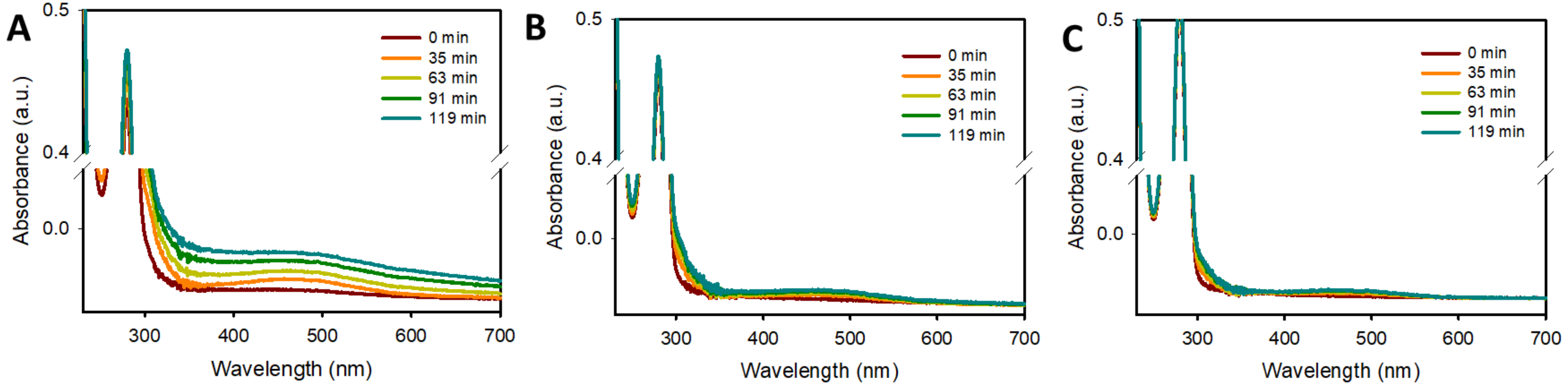

Figure S7. Effect of pH on the degradation of DA. (**A-C**) Temporal variation of the UV-Vis absorbance spectra at 37°C of solutions containing DA at 100 μM concentration prepared in a 10 mM phosphate solution containing 75 mM NaCl at pH 7.4 (**A**), pH 6.5 (**B**) and pH 6.0 (**C**).

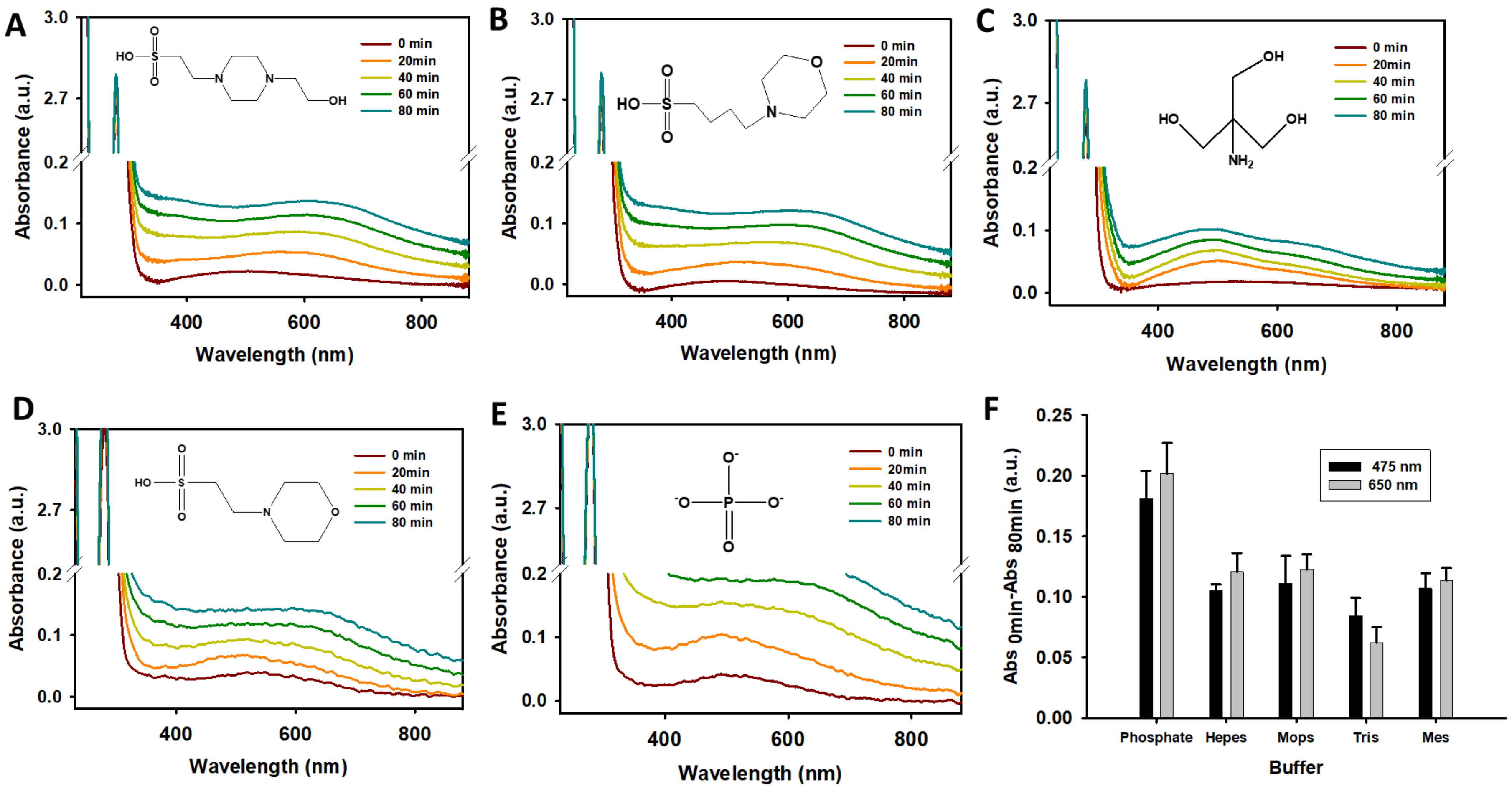

Figure S8. Effect of the buffer type on the Fe^{3+}-catalyzed degradation of DA. (**A-C**) Temporal variation of the UV-Vis absorbance spectra of DA solutions (1 mM) prepared in different buffer solutions containing 75 mM NaCl and 10 µM Fe^{3+}. The incubations were carried out at 37°C and at pH 7.4, and the buffers used in this study were 20 mM HEPES (**A**), 20 mM MOPS (**B**), 20 mM TRIS (**C**), 20 mM MES (**D**) and 20 mM phosphate (**E**). The structural formulate of the different buffers are shown as insert on each graph. (**F**) Differences in the absorbance at 475 nm (*black*) and at 650 nm (*grey*) between the UV spectra of DA (1 mM) obtained after 0 and 80 minutes of incubation in each buffer (i.e. phosphate, HEPES, MOPS, TRIS and MES) at pH 7.4 and 37°C.

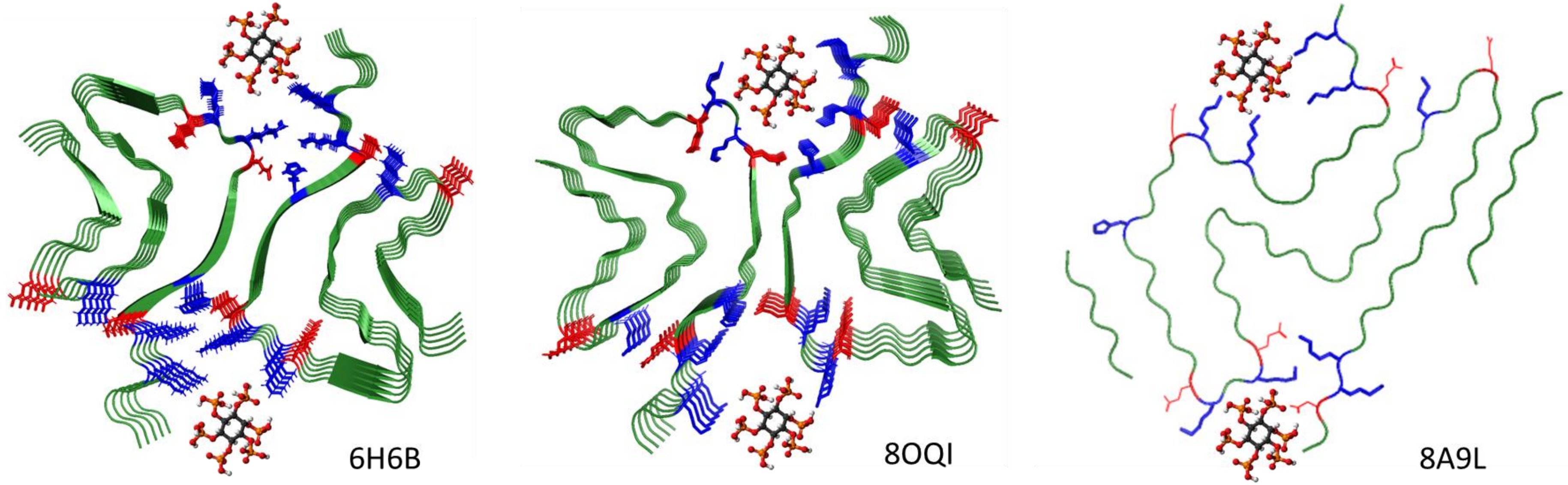

Figure S9. Three dimensional structures of αS amyloid protofibrils. **(Left)** Cryo-EM structure (PDB code 6H6B) of a wild-type αS amyloid fibril assembled by two protofibrils. **(Middle)** Cryo-EM structure (PDB code 8OQI) of a different wild-type αS amyloid fibril also assembled by two protofibrils. **(Right)** Cryo-EM structure of αS filaments isolated from Lewy bodies (PDB code 8A9L). The backbone of αS in the structures of the different fibrils is shown in green, whereas the side chains of the cationic (Lys, Arg and His) and anionic (Asp and Glu) residues are shown in sticks. The cationic residues are coloured in blue, whereas the anionic residues are coloured in red. A molecule of IP6 (in ball and sticks) has been located in the cationic pocket of the fibril, assuming that this would be the most likely binding pose for the anionic IP6.
